# Blood methylome signatures in children exposed to maternal type 1 diabetes are linked to protection against islet autoimmunity

**DOI:** 10.1038/s42255-025-01403-w

**Published:** 2025-11-06

**Authors:** Raffael Ott, Jose Zapardiel-Gonzalo, Peter Kreitmaier, Kristina Casteels, Angela Hommel, Olga Kordonouri, Helena Elding Larsson, Agnieszka Szypowska, Manu Vatish, Eleftheria Zeggini, Annette Knopff, Christiane Winkler, Anette-G. Ziegler, Ezio Bonifacio, Sandra Hummel

**Affiliations:** 1 Institute of Diabetes Research, Helmholtz Munich, German Research Center for Environmental Health, Neuherberg, Germany; 2 Technical University of Munich, School of Medicine and Health, Forschergruppe Diabetes, TUM University Hospital, Munich, Germany; 3 Forschergruppe Diabetes e.V. at Helmholtz Zentrum München, Munich, Germany; 4 Institute of Translational Genomics, Helmholtz Zentrum München—German Research Center for Environmental Health, Neuherberg, Germany; 5 Technical University of Munich (TUM), TUM University Hospital, TUM School of Medicine and Health, Munich, Germany; 6 Department of Pediatrics, University Hospital Gasthuisberg, Leuven, Belgium; 7 Department of Development and Regeneration, KU Leuven, Leuven, Belgium; 8 Center for Regenerative Therapies Dresden, Faculty of Medicine, Technische Universität Dresden, Dresden, Germany; 9 Paul Langerhans Institute Dresden of Helmholtz Munich at University Hospital Carl Gustav Carus, Faculty of Medicine, Technische Universität Dresden, Dresden, Germany; 10 Kinder- und Jugendkrankenhaus AUF DER BULT, Hannover, Germany; 11 Unit for Pediatric Endocrinology, Department of Clinical Sciences Malmö, Lund University, Lund, Sweden; 12 Department of Pediatrics, Skane University Hospital, Malmö, Sweden; 13 Department of Pediatric Diabetology and Pediatrics, The Children’s Clinical Hospital Józef Polikarp Brudziński, University Clinical Centre of the Medical University of Warsaw, Warsaw, Poland; 14 Department of Pediatric Diabetology and Pediatrics, Medical University of Warsaw, Warsaw, Poland; 15 Nuffield Department of Women’s and Reproductive Health, University of Oxford, Oxford, UK; 16 John Radcliffe Hospital, Oxford, UK; 17 German Center for Diabetes Research (DZD), Neuherberg, Germany; 18 Present Address: German Center for Diabetes Research (DZD), Neuherberg, Germany

**Keywords:** Type 1 diabetes, Epigenomics, Metabolism

## Abstract

Exposure to maternal type 1 diabetes (T1D) during pregnancy provides relative protection against T1D in the offspring. This protective effect may be driven by epigenetic mechanisms. Here we conducted an epigenome-wide blood analysis on 790 young children with and 962 children without a T1D-affected mother, and identified differential DNA methylation (*q* < 0.05) at multiple loci and regions. These included the Homeobox A gene cluster and 15 T1D susceptibility genes. The differential methylation was found in transcriptionally relevant regions associated with immune function, including sites previously linked to T1D-related methylation loci and protein biomarkers. Propensity scores for methylation at T1D susceptibility loci could predict the development of islet autoimmunity in offspring born to mothers without T1D. Together, these findings highlight pathways through which maternal T1D may confer protection against islet autoimmunity in offspring and suggest that environmental factors can influence T1D risk through epigenetic modifications of T1D susceptibility loci.

## Abstract (web summary)

Through an epigenome-wide blood analysis in children of mothers with or without type 1 diabetes, the authors identify epigenetic modifications of type 1 diabetes susceptibility loci through which maternal type 1 diabetes may protect from islet autoimmunity in offspring.

## Main

Early-life development is highly susceptible to environmental influences, which can have enduring impacts on health^1^. Epigenetic mechanisms, such as DNA methylation, play a pivotal role in mediating the effects of environmental factors on phenotypic traits^2^. Furthermore, it is increasingly evident that a mother’s lifestyle and clinical condition can have intergenerational effects, which are possibly mediated through epigenetic alterations^3,4^. For example, numerous studies have demonstrated that maternal smoking during pregnancy^4,5^, maternal stress^6^, prepregnancy body mass index^7^ and maternal diet^3,8^ are associated with DNA methylation changes in young offspring.

Type 1 diabetes (T1D) is among the most common chronic diseases in childhood and adolescence^9^, and results from autoimmune destruction of the insulin-producing beta cells in pancreatic islets. The clinical onset of T1D typically follows a presymptomatic stage, detectable through the presence of autoantibodies against multiple islet autoantigens, which often arise in the first years of life^10^. Genetic susceptibility, particularly involving immune response genes, is well-established, and gene‒environment interactions are believed to initiate the autoimmune process^11,12^. The risk of developing T1D is 8‒15 times higher in individuals who have a first-degree relative with T1D than in individuals with no family history of T1D^13^. This risk further varies on the basis of whether the affected family member is a mother, sibling or father^13^. Consistent with the intergenerational effects of maternal T1D, there is strong evidence for substantially lower risk in the offspring of mothers with T1D than in offspring with an unaffected mother but an affected father or sibling^13^. Similarly, the likelihood of early development of islet autoantibodies is lower in the offspring of mothers with T1D, particularly within the first 2 years of life^14^. Various mechanisms have been suggested to explain this relative protection, including the transplacental transfer of maternal islet autoantibodies^15^, enhanced immune regulation against islet beta cell autoantigens^16^ and direct effects of hyperglycaemia on pancreatic islet development^17^. However, the underlying molecular mechanisms are not understood, and the impact of maternal T1D on early-life DNA methylation, as well as the potential protective epigenetic mechanisms, remain to be explored.

In this study, we proposed that the offspring of mothers with T1D would exhibit differential methylation of cytosine–phosphate–guanine (CpG) dinucleotides, some of which affect T1D susceptibility. To investigate this, we conducted an epigenome-wide association study (EWAS) of blood samples from prospective birth cohorts of children at increased risk of T1D and examined the differentially methylated CpGs in offspring of mothers with T1D for their associations with T1D susceptibility.

## Results

### Study cohorts

**Extended Data Fig. 1:**
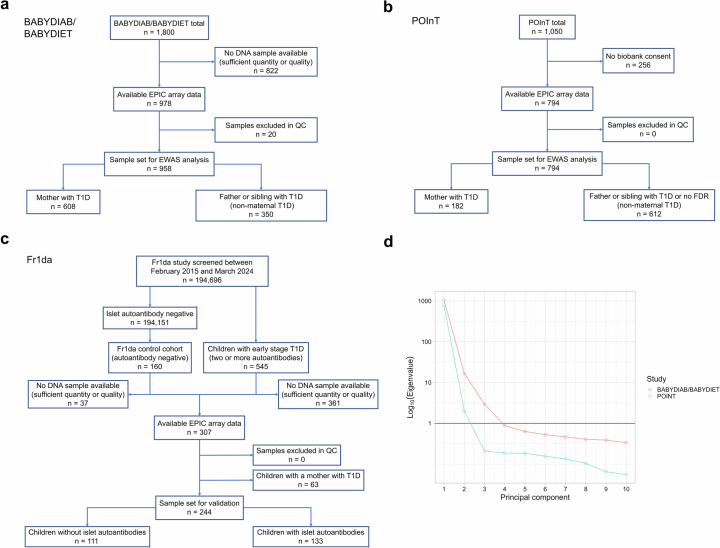
Flow-chart of children included in this study and scree plot of principal components. **a-c**, Flow-charts of the children participating in the BABYDIAB/BABYDIET (**a**), POInT (**b**) and Fr1da (**c**) study included in this analysis. **d**, Scree plot of eigenvalues as logarithmic scale of the first ten principal components derived from principal component analysis (PCA) using all 651,271 CpG sites in the BABYDIAB/BABYDIET and POInT studies. The first three principal components covered over 95% of variability and were included in the linear models. An eigenvalue of one is indicated by the horizontal line. QC: Quality control; EWAS: Epigenome-wide association study; T1D: Type 1 diabetes; FDR: First-degree relative. Source data

We used the Illumina EPIC array to determine the methylation status of blood DNA samples collected from 1,752 children participating in two prospective longitudinal studies, BABYDIAB/BABYDIET and Primary Oral Insulin Trial (POInT), including 790 children who had a mother with T1D (Supplementary Table 1 and Extended Data Fig. 1a,b). All children had an increased risk of T1D, defined by a first-degree family history^18,19^ or genetic risk score^20^, compared with children of the background population. The median age at sample collection was 2.1 years (interquartile range (IQR) 1.9‒2.4 years) in the BABYDIAB/BABYDIET cohort and 1.50 years (IQR 1.49‒1.52 years) in the POInT cohort. The calculated methylation age of samples was around 0.5 years lower than actual age in both studies and showed no difference between the children with versus without a mother with T1D (Supplementary Table 1). The EWAS analyses were adjusted for age at DNA sample collection, sex, technical variables and six estimated blood cell types. The estimated blood cell types showed no difference in frequency between children with a mother with T1D and children with an unaffected mother (Supplementary Table 1). To improve the robustness of the analysis, we removed CpGs with high heterogeneity (*I*^2^ > 75) between the studies (Methods).

### Maternal T1D and DNA methylome in the offspring

**Fig. 1:**
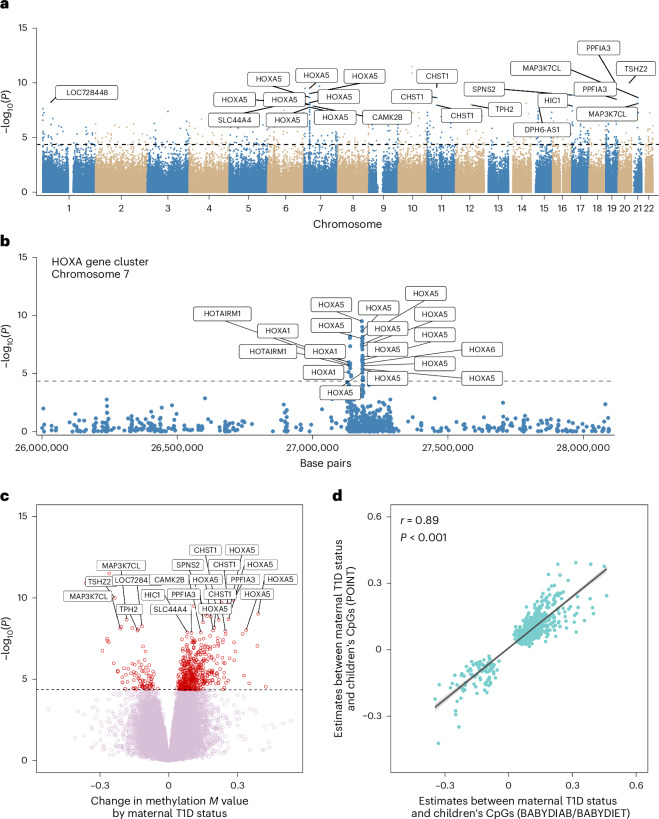
Epigenome-wide blood DNA methylation differences at CpG sites between children born to a mother with and without T1D. We analysed the methylation status of 651,271 CpG sites in children with (*n* = 790) and without a mother with T1D (*n* = 962). **a**, Manhattan plot showing the −log_10_(*P*) of the associations between the children’s blood DNA methylation status of individual CpG sites across the autosomal chromosomes (1‒22) and maternal T1D status (adjusted linear regression, two-sided, multiple testing correction using FDR). **b**, Higher-magnification view of **a** including the HOXA gene cluster at chromosome 7p15 (adjusted linear regression, two-sided, multiple testing correction using FDR). **c**, Volcano plot showing −log_10_(*P*) against the effect estimates of the CpG analysis (change in methylation *M* value by maternal T1D; adjusted linear regression, two-sided, multiple testing correction using FDR). **d**, Correlation (Pearson’s *r*, one-sided) between effect estimates of the 566 CpGs associated with maternal T1D in the individual studies. Lines were fit using a linear model and the range of uncertainty is given as the 95% confidence intervals (highlighted in grey). Each dot represents a CpG site. Horizontal dashed lines indicate the *P*_FDR_ threshold. The annotated genes of the top CpGs are displayed. Source data

A total of 566 differentially methylated CpG sites were identified in the offspring of mothers with T1D (*P*_FDR_ < 0.05; Fig. 1a and Supplementary Table 2). Thirty-three of the 566 CpGs were annotated to the Homeobox A (HOXA) gene cluster at chromosome 7p15, particularly *HOXA5* (Fig. 1b). The HOXA proteins belong to a large class of transcription factors with essential roles in early-life development and organogenesis^21^. HOXA5 has been linked to adiposity^22^ and inflammatory processes^22,23^. Most (478 of 566) of the identified CpG sites exhibited hypermethylation in the offspring of mothers with T1D (Fig. 1c). The effect estimates of the differentially methylated CpGs were strongly correlated between the BABYDIAB/BABYDIET and POInT studies when analysed separately (Pearson’s *r* = 0.89, *P* < 0.001; Fig. 1d). Sex-specific analysis revealed a strong correlation of the effect estimates of the 566 CpGs between female and male offspring (Pearson’s *r* = 0.91, *P* < 0.001).

**Fig. 2:**
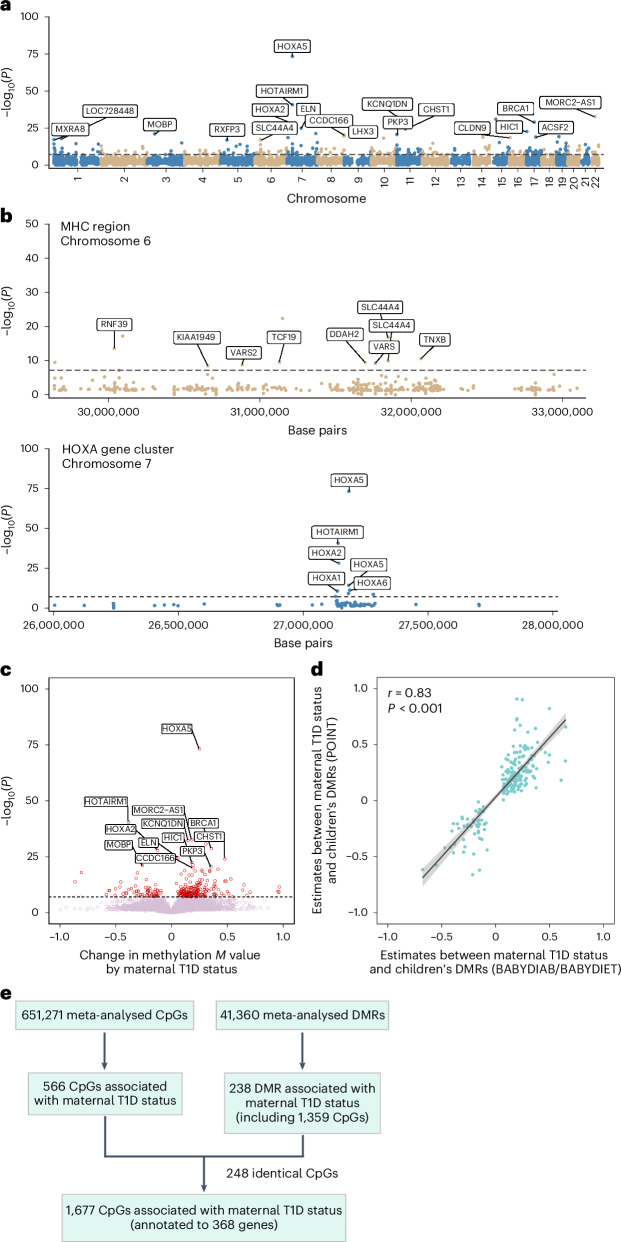
Epigenome-wide blood DNA methylation at DMRs between children born to a mother with and without T1D. We analysed the methylation status of 41,360 genomic regions in children with (*n* = 790) and without a mother with T1D (*n* = 962). **a**, Manhattan plot showing the −log_10_(*P*) of the associations between the children’s DMRs and maternal T1D (adjusted linear regression, two-sided, multiple testing correction using FDR). **b**, Higher-magnification view of **a** including the MHC region at chromosome 6p21 (upper panel) and the HOXA gene cluster at chromosome 7p15 (lower panel; adjusted linear regression, two-sided, multiple testing correction using FDR). **c**, Volcano plot showing −log_10_(*P*) against the effect estimates of the DMRs analysis (change in methylation *M* value by maternal T1D; adjusted linear regression, two-sided, multiple testing correction using FDR). **d**, Correlation (Pearson’s *r*, one-sided) between effect estimates of the DMRs associated with maternal T1D in the individual studies. Lines were fit using a linear model and the range of uncertainty is given as the 95% confidence intervals (highlighted in grey). **e**, Summary of the epigenome-wide CpG and region analyses. **a**–**e**, Each dot represents a methylated region. Horizontal dashed lines indicate the *P*_FDR_ threshold. The annotated genes of the top DMRs are displayed. Source data

Individual CpG sites in proximity often exhibit similar methylation patterns. Therefore, we investigated 41,360 methylated regions using the dmrff R package, which generates regions using consecutive CpGs that are at most 500 base pairs apart on the basis of nominal significance (*P* < 0.05) and the same effect size direction in the meta-EWAS. Of the 41,360 generated regions, 238 regions were DMR in the offspring of mothers with T1D (*P*_FDR_ < 0.05; Fig. 2a and Supplementary Table 2). These encompassed 1,359 differentially methylated CpGs, including 248 of the 566 CpGs identified in the preceding analysis, and were associated with the major histocompatibility complex (MHC) on chromosome 6p21, known to confer the major genetic susceptibility for and resistance to T1D^12^ and the HOXA gene cluster (Table 1 and Fig. 2b). A predominant hypermethylation (178 of 238 DMRs) was detected in the offspring of affected mothers (Fig. 2c). Again, there was a strong correlation of the effect estimates between the individual studies when analysed separately (Pearson’s *r* = 0.83, *P* < 0.001; Fig. 2d). When analysed separately by sex, the effect estimates of the 238 DMRs in female and male offspring were highly correlated (Pearson’s *r* = 0.90, *P* < 0.001).

**Table 1:** Top 20 DMRs in children’s blood associated with maternal T1D based on *P*_FDR_

Genomic region of the DMR (chr.: start–end)^a^	Annotated gene	Estimate (s.e.)	*P*_FDR_
7:27183274–27185732	*HOXA5/HOXA6*	0.25 (0.01)	5.0 × 10^−68^
7:27137427–27138974	*HOTAIRM1*	–0.39 (0.03)	2.0 × 10^−35^
22:31317914–31318546	*C22orf27*	0.18 (0.02)	1.8 × 10^−27^
11:2890319–2890710	*KCNQ1DN*	0.15 (0.01)	3.7 × 10^−27^
15:31515750–31516404		0.30 (0.03)	1.2 × 10^−25^
17:41278179–41278622	*BRCA1/NBR2*	0.35 (0.03)	1.2 × 10^−23^
7:27142100–27143788	*HOXA2*	–0.13 (0.01)	6.0 × 10^−23^
7:73441989–73442531	*ELN*	0.06 (0.01)	1.3 × 10^−19^
11:45671208–45671369	*CHST1*	0.47 (0.05)	5.2 × 10^−19^
17:1961286–1961778	*HIC1*	0.18 (0.02)	2.4 × 10^−17^
6:31148332–31148748		0.18 (0.02)	3.3 × 10^−17^
7:150870852–150872218		0.19 (0.02)	5.3 × 10^−16^
3:39543544–39544326	*MOBP*	–0.26 (0.03)	1.0 × 10^−15^
11:396686–397486	*PKP3*	0.35 (0.04)	2.7 × 10^−15^
8:144790089–144790729	*CCDC166*	0.19 (0.02)	6.6 × 10^−15^
8:144787999–144789164		0.41 (0.05)	3.3 × 10^−14^
19:12305553–12305869		0.27 (0.03)	7.1 × 10^−14^
9:139092501–139093499	*LHX3*	0.34 (0.04)	1.1 × 10^−13^
17:48545571–48547118	*CHAD/ACSF2*	0.11 (0.01)	1.3 × 10^−13^
14:70316670–70317582		0.21 (0.02)	2.0 × 10^−13^

^a^Human genome assembly GRCh37/hg19.

Adjusted linear regression analysis (two-sided) with multiple testing correction using FDR. chr.: chromosome; s.e.: standard error.

**Extended Data Fig. 2:**
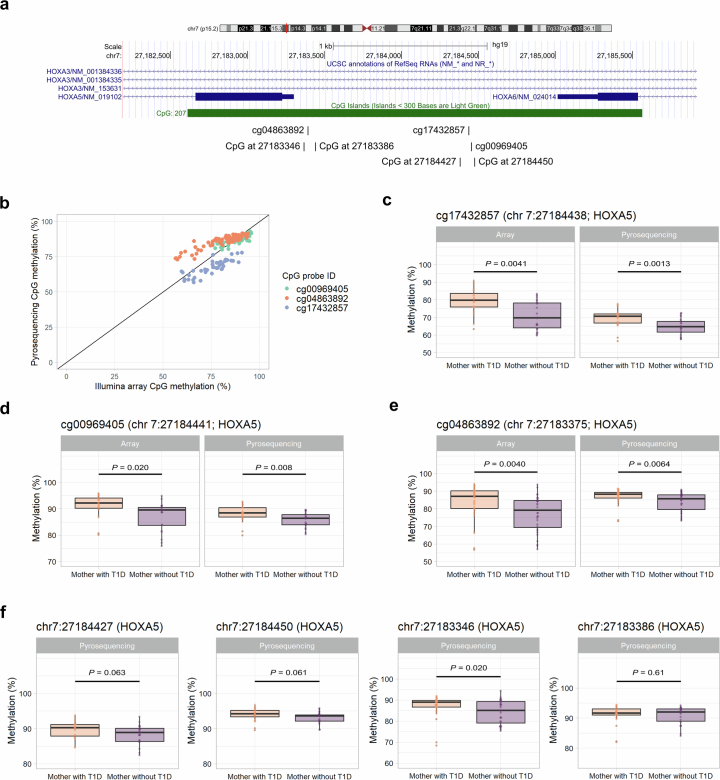
Validation of maternal T1D offspring-associated CpGs at *HOXA5.* **a**, UCSC Genome Browser map (hg19) of the *HOXA5* and *HOXA6* promoter regions with indicated locations of the presented CpGs at *HOXA5*. **b**, Correlation plot showing blood methylation values of maternal T1D associated CpGs at *HOXA5* between the Illumina EPIC array and bisulfite pyrosequencing in children (*n* = 45-73). **c-e**, Methylation levels at maternal T1D associated CpGs in children with mothers with T1D vs. children with unaffected mothers determined by Illumina EPIC array or bisulfite pyrosequencing (c: *n* = 46, d: *n* = 45, e: *n* = 73). **f**, Methylation levels of CpGs near the Illumina EPIC array *HOXA5* probes in children with mothers with T1D vs. children with unaffected mothers determined by bisulfite pyrosequencing (left: *n* = 48, middle left: *n* = 48, middle right: *n* = 74, right: *n* = 52). Box-plots indicate the median and the 25^th^ and 75^th^ percentile, whiskers extend 1.5 times the IQR from the top and bottom of the box. Uncorrected *P*-values derived from Welch’s t-test or Mann-Whitney-U test (two-sided) are presented. Source data

**Extended Data Fig. 3:**
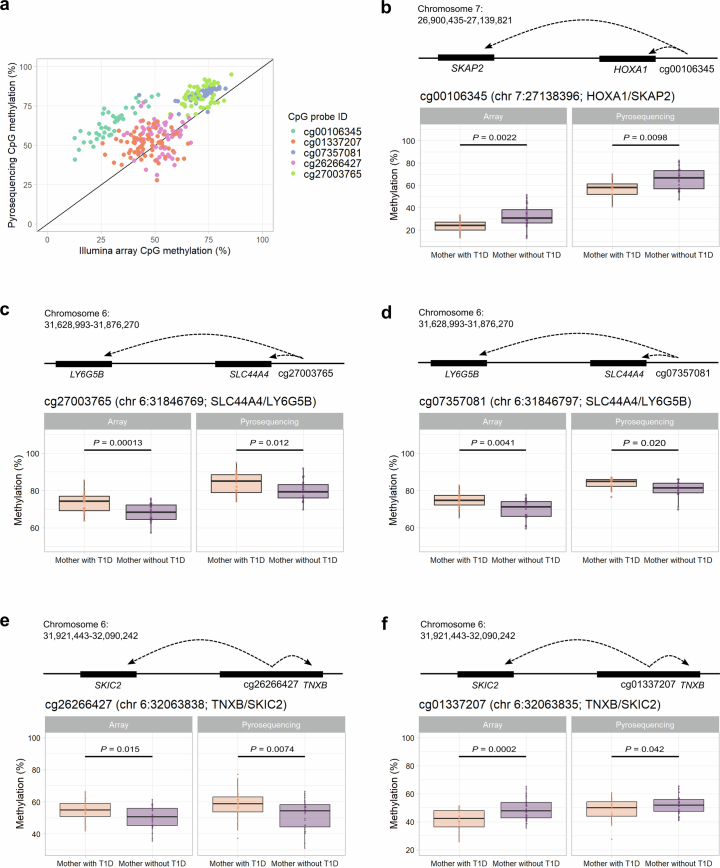
Validation of maternal T1D offspring-associated CpGs at T1D susceptibility genes. **a**, Correlation plot showing blood methylation values of maternal T1D associated CpGs linked to T1D susceptibility genes between the Illumina EPIC array and bisulfite pyrosequencing in young children (*n* = 40-67). **b-f**, Methylation levels at CpGs linked to T1D susceptibility genes (*SKAP2*, *SLC4A44*, *LY6G5B*, *TNXB*, *SKIC2*) in children with mothers with T1D vs. children with unaffected mothers determined by Illumina EPIC array or bisulfite pyrosequencing (b: *n* = 40, c: *n* = 57, d: *n* = 40, e: *n* = 63, f: *n* = 67). A schematic regulatory map is shown above each CpG based on the eQTM-derived CpG target genes. Box-plots indicate the median and the 25^th^ and 75^th^ percentile, whiskers extend 1.5 times the IQR from the top and bottom of the box. Uncorrected *P*-values derived from Welch’s t-test or Mann-Whitney-U test (two-sided) are presented. Source data

Differences at three CpGs and four neighbouring CpGs of the *HOXA5* promoter region and at five CpGs linked to five T1D susceptibility genes were validated by bisulfite pyrosequencing (Extended Data Figs. 2 and 3).

**Extended Data Fig. 4:**
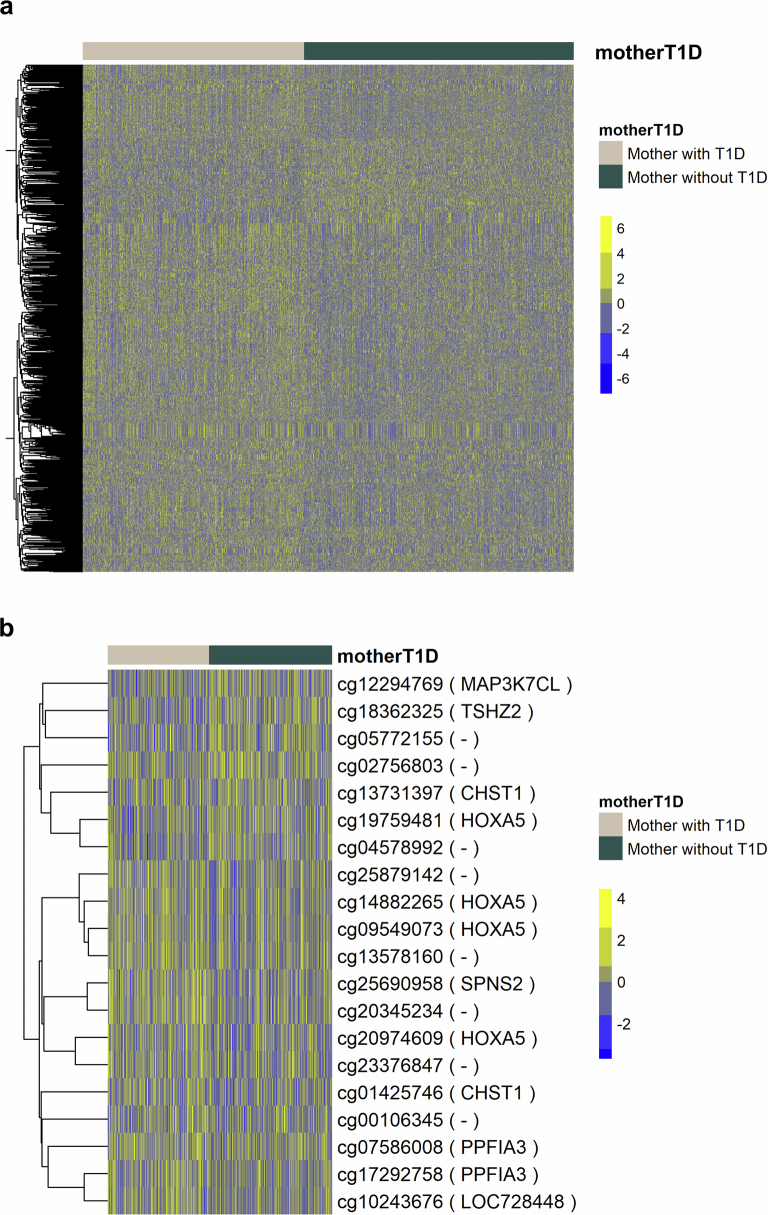
Heatmaps of all and top 20 maternal T1D offspring-associated CpG sites. **a**, Heatmap of adjusted and scaled methylation M-values of 1,752 children across the 1,677 CpG sites associated with maternal T1D. **b**, Heatmap of adjusted and scaled methylation M-values of the 20 most significant CpGs of 1,752 children. Illumina probe IDs and annotated genes are shown aside each row. CpG clustering was performed based on Euclidean distance. Source data

In total, 1,677 CpGs with differential methylation between the offspring of mothers with T1D and the offspring without an affected mother were identified for downstream analyses (Fig. 2e, Extended Data Fig. 4 and Supplementary Table 2).

Of these, only 14 (0.8%) were associated with factors linked to or affected by maternal T1D that were available in the cohorts: 8 CpGs were associated with maternal age at pregnancy and 6 associated with birth weight (Supplementary Table 2). No associations were found for parity, delivery by Caesarean section or maternal glycated HbA1c during the last trimester of pregnancy.

A comparison of the maternal T1D-associated loci with differential blood methylation and transcription in much older offspring of mothers with T1D^24^ revealed no overlapping CpG site or DMR, but some similar gene targets, including *Potassium voltage-gated channel subfamily Q member 2* (*KCNQ2*) on chromosome 20q13.33, *Caldesmon 1* (*CALD1*) on chromosome 7q33 and *Chitinase domain containing 1* (*CHID1*) on chromosome 11p15.5.

### Differentially methylated CpG enrichment at regulatory regions

**Fig. 3:**
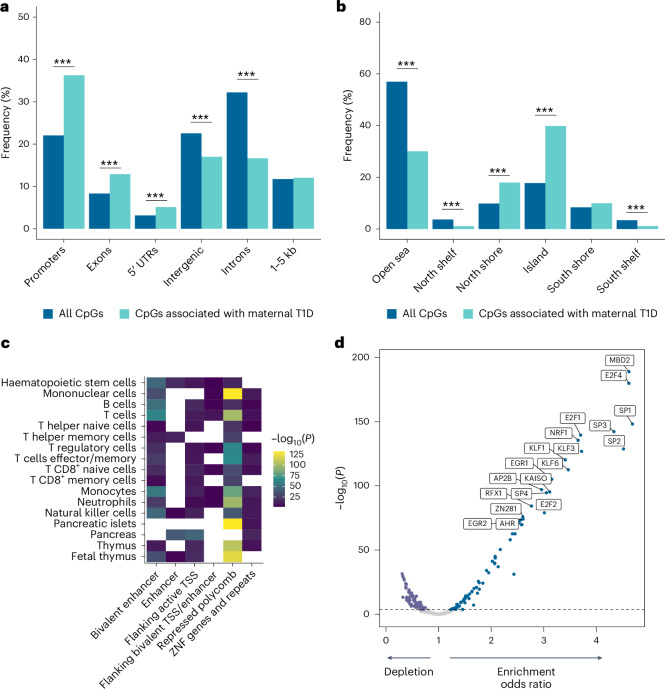
Maternal T1D offspring-associated CpGs are enriched at gene regulatory regions. **a**, Frequency of 1,677 maternal T1D offspring-associated CpG locations in major gene regions compared with all CpGs included in the meta-analyses (hypergeometric test, promoters *P* = 3.8 × 10^−40^, exons *P* = 1.5 × 10^−10^, 5′ untranslated region (UTR) *P* = 1.2 × 10^−5^, intergenic *P* = 1.2 × 10^−8^, introns *P* = 1.0 × 10^−47^, multiple testing correction using Bonferroni). **b**, Frequency of 1,677 maternal T1D offspring-associated CpG locations according to CpG island categories compared with all CpGs included in the meta-analyses (hypergeometric test, open sea *P* = 2.0 × 10^−110^, north shelf *P* = 8.6 × 10^−12^, north shore *P* = 2.3 × 10^−24^, island *P* = 8.1 × 10^−100^, south shelf *P* = 1.2 × 10^−9^, multiple testing correction using Bonferroni). **c**, Enrichments for regulatory elements of 1,677 maternal T1D offspring-associated CpG locations across specific immune cell types and tissues with relevance for T1D compared with all CpGs included in the meta-analysis (white tiles represent no enrichments; hypergeometric test, one-sided, multiple testing correction using Bonferroni). **d**, Transcription factor binding motif analysis of 1,677 maternal T1D offspring-associated CpGs (Fisher’s exact test, two-sided, multiple testing correction using FDR). The dashed line indicates the *P*_FDR_ threshold. The most enriched transcription factor binding motifs are highlighted. TSS, transcription start site; ZNF, zinc-finger proteins. ***P < 0.001 (hypergeometric test, one-sided, multiple testing correction using Bonferroni). Source data

We examined the genomic locations of the 1,677 differentially methylated CpGs in the offspring of mothers with T1D (Fig. 3). Enrichments were found at gene regions essential for transcriptional regulation, such as the promoter (hypergeometric test, *P* = 3.8 × 10^−40^), 5′ untranslated region (*P* = 1.2 × 10^−5^; Fig. 3a) and CpG islands (*P* = 8.1 × 10^−100^; Fig. 3b). We used publicly available chromatin marks characteristic for regulatory elements in specific immune cell types and T1D-relevant tissues from the Roadmap Consortium to examine enrichments of regulatory elements among the 1,677 CpGs (Fig. 3c). Enrichments were found across multiple blood cell types for the same regulatory elements, such as bivalent enhancers, contributing to active or repressive transcription and Polycomb repressive marks, contributing to inhibition of transcription (Fig. 3c). Of note, regulatory elements, such as enhancer and Polycomb repressive marks, were also enriched in relevant tissues of T1D development, for example, the pancreas and thymus (Fig. 3c).

Motif enrichment analysis of transcription factor binding sites identified 94 enriched motifs (Fisher’s exact test, *P*_FDR_ < 0.05; Fig. 3d). The transcriptional repressor Methyl-CpG binding domain protein 2 (MBD2) showed the strongest enrichment (*P*_FDR_ = 3.9 × 10^−187^; Fig. 3d). MBD2 is essential for T cell development^25,26^ and differentiation^27^, and is important for regulatory T cell (T_reg_) function^28^. T cells are the key mediators of the autoimmune processes leading to the destruction of pancreatic beta cells in T1D^29,30^. MBD2 deficiency has been shown to exacerbate the development of autoimmune diabetes in non-obese diabetic mice^27^.

Taken together, these findings show that exposure to maternal T1D was predominantly associated with methylation sites at transcriptionally relevant regions that are involved in immune cell function and the development of autoimmunity.

### Methylome differences and T1D risk genes and biomarkers

We examined the potential effects of the differentially methylated CpGs on the expression of genes and proteins associated with susceptibility for T1D using publicly available *cis*-expression quantitative trait methylation (eQTM) and protein quantitative trait methylation data.

**Fig. 4:**
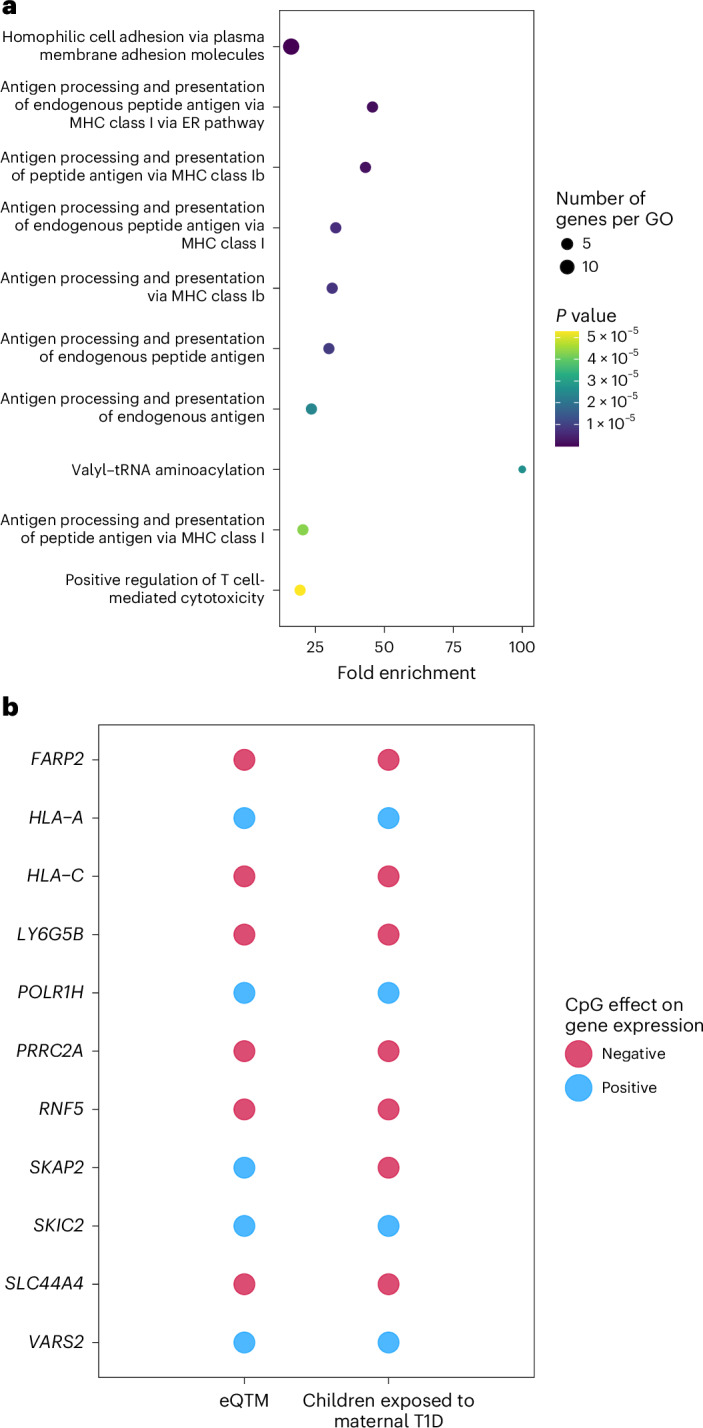
Identified CpGs target T1D susceptibility genes according to eQTM data of children’s blood. **a**, Gene ontology (GO) analysis of 142 eQTM gene targets of the 1,677 maternal T1D offspring-associated CpGs. Only the top ten ontologies are shown (Fisher’s exact test, one-sided, multiple testing correction using FDR). ER, endoplasmic reticulum. **b**, Observed effects of CpGs (in terms of increase in methylation) on the expression of 11 T1D susceptibility genes (positive or negative association) according to *cis*-eQTM data and effects on gene expression based on changes in methylation of the corresponding CpGs associated with exposure to maternal T1D (Supplementary Table 3). Source data

We observed associations with RNA transcripts of 142 unique genes (Supplementary Table 3). The gene ontologies of these eQTM-derived genes indicated over-representation for 20 terms (Fisher’s exact test, *P*_FDR_ < 0.05; Fig. 4a), including eight that are directly related to immune system function, particularly peptide processing in MHC human leukocyte antigen (HLA) class I, which is relevant to T1D susceptibility^31^. Notably, the 142 genes included the chromosome 7p15 HOXA genes and 11 T1D susceptibility genes. These were *FERM*, *RhoGEF* and *pleckstrin domain protein 2* (*FARP2*) on chromosome 2q32 (ref. ^32^), *Src kinase associated phosphoprotein 2* (*SKAP2*) on chromosome 7p15 (ref. ^32^) and nine genes located at the MHC region on chromosome 6p21 (*HLA-A*, *HLA-C*, *LY6G5B*, *POLR1H*, *PRRC2A*,*RNF5*, *SLC44A4*, *SKIC2* and *VARS2*)^32–34^. The differences in methylation seen between the offspring of mothers with and without T1D are consistent with reduced transcription of *FARP2*, *HLA-C*, *LY6G5B*, *PRRC2A*, *RNF5*, *SKAP2* and *SLC44A4* and increased expression of *HLA-A*, *POLR1H*, *SKIC2* and *VARS2* (Fig. 4b and Supplementary Table 3). Among the annotations of the 1,677 CpGs, four additional candidate T1D susceptibility genes (*FUT2*, *IL27*, *PPP1R18*, *TNXB*)^32,34^ were identified. In total, 105 differentially methylated CpGs were identified in the offspring of mothers with T1D that had potentially regulatory effects on 15 T1D susceptibility genes, including 2 HLA-related genes and 7 non-HLA genes located within the MHC region of chromosome 6p21.

We also examined associations with protein traits and found 198 of the differentially methylated CpGs in the offspring of mothers with T1D to be associated with 239 circulating protein traits. These included 14 proteins that were previously identified as candidate biomarkers for T1D (Supplementary Table 4). Notably, we observed over-representation of 52 CpGs with circulating Cysteine rich secretory protein 2 (CRISP2) protein levels (hypergeometric test, *P* = 1.4 × 10^−54^). The concentration of CRISP2 is associated with progression to T1D^35^ and the *CRISP2* gene was shown to be differentially expressed in regulatory T lymphocytes from patients with T1D^36^.

We further assessed the overlap of the differentially methylated CpGs and DMRs with blood methylation sites and regions related to T1D development in previous studies. We identified eight CpGs and six DMRs (including 37 CpGs) among the differentially methylated loci and regions that were previously related to T1D (Supplementary Table 5). Of these, five CpGs and six DMRs showed differences in children before the onset of T1D, including two CpGs and two DMRs already differentially methylated before islet autoantibody seroconversion (Supplementary Table 5).

Therefore, the differentially methylated CpGs in the offspring of mothers with T1D compared with offspring with unaffected mothers show notable associations with the genes, proteins and methylation loci involved in T1D susceptibility.

### Maternal T1D offspring methylation score and islet autoimmunity

**Fig. 5:**
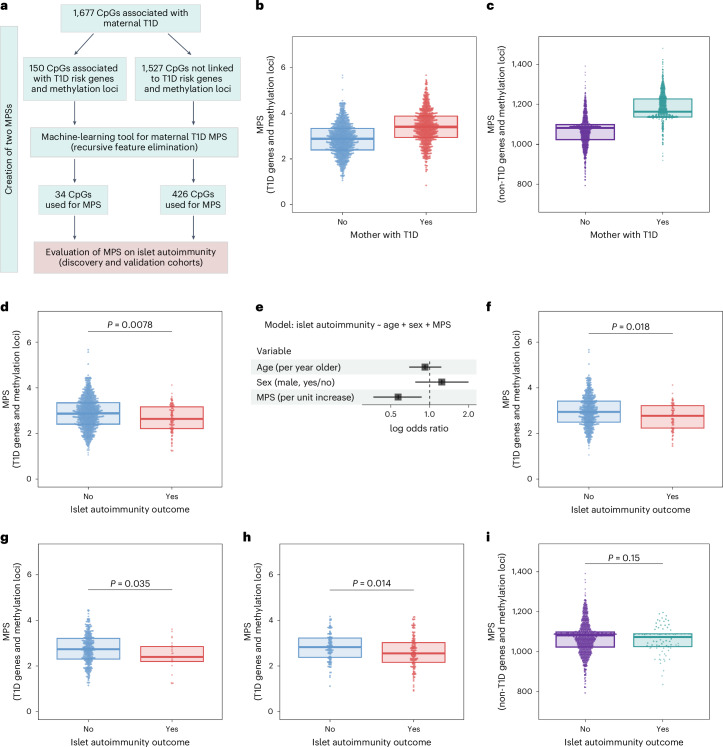
Maternal T1D-MPS links combined CpG methylation to islet autoimmunity. **a**, The main steps in generating the two MPSs. **b**, The MPS based on 34 CpGs linked to T1D susceptibility loci by maternal T1D status (*n*_total_ = 1,752, *n* children with a mother with T1D = 790). **c**, The MPS based on 426 CpGs not linked to T1D susceptibility loci by maternal T1D status (*n*_total_ = 1,752, *n* children with a mother with T1D = 790). **d**, The MPS based on 34 CpGs linked to T1D susceptibility loci in children not exposed to maternal T1D by islet autoimmunity outcome (*n*_total_ = 962, *n* = 81 children who developed islet autoimmunity; adjusted logistic regression, two-sided). **e**, Forest plot of the variables used in logistic regression models between the MPS based on 34 CpGs linked to T1D susceptibility loci and the islet autoimmunity outcome in children not exposed to maternal T1D (*n*_total_ = 962, *n* = 81 children who developed islet autoimmunity; adjusted logistic regression, two-sided). The derived odds ratios with corresponding 95% confidence intervals are shown on a log scale. **f**, The MPS based on 34 CpGs linked to T1D susceptibility loci in children with a father and/or sibling with T1D by islet autoimmunity outcome (*n*_total_ = 603, *n* = 58 children who developed islet autoimmunity, adjusted logistic regression, two-sided). **g**, The MPS based on 34 CpGs linked to T1D susceptibility loci in children without a first-degree relative with T1D by islet autoimmunity outcome (*n*_total_ = 359, *n* = 23 children who developed islet autoimmunity, adjusted logistic regression, two-sided). **h**, Validation of the MPS based on 34 CpGs linked to T1D susceptibility loci in children without a mother with T1D from an independent cohort (Fr1da) by islet autoimmunity outcome (*n*_total_ = 244, *n* = 133 children who developed islet autoimmunity, adjusted logistic regression, two-sided). **i**, The MPS based on 426 CpGs not linked to T1D susceptibility loci in children not exposed to maternal T1D by islet autoimmunity outcome (*n*_total_ = 962, *n* = 81 children who developed islet autoimmunity, adjusted logistic regression, two-sided). Box-plots indicate the median and the 25th and 75th percentiles of the individual samples (dots). *P* values derived from adjusted logistic regression (two-sided). Source data

A feature of maternal T1D protection against islet autoimmunity is that it is relative to paternal T1D and, therefore, operates on an a priori T1D susceptible genetic background. As a consequence, a key question is whether the differentially methylated CpGs in the offspring of mothers with T1D, particularly CpGs linked to T1D susceptibility loci, are directly associated with protection against islet autoimmunity. To address this, we separately examined (1) the differentially methylated CpGs assigned to the 15 T1D susceptibility genes (105 CpGs; Supplementary Table 6) and the 45 CpGs previously related to T1D (Supplementary Table 6), and (2) the 1,527 differentially methylated CpGs without known links to T1D susceptibility genes or methylation loci. For each of these CpG sets, we constructed a methylation propensity score (MPS) for offspring of mothers with T1D using recursive feature elimination, a machine-learning tool used to generate methylation scores^37^. This was done to identify the optimal CpG set that captures the effect of maternal T1D on CpG methylation in their offspring (Fig. 5a). The optimal weighted scores included 34 CpGs for the T1D susceptibility loci set (Supplementary Table 6), including 28 CpGs linked to T1D susceptibility genes, and 426 CpGs for the non-T1D susceptible loci set (Supplementary Table 6). The MPS for the T1D susceptible loci CpG set (area under the receiver-operating curve 0.71) and the MPS for the non-susceptible loci CpG set (area under the receiver-operating curve 0.88) effectively reflected the maternal T1D status of the offspring (Fig. 5b,c).

**Extended Data Fig. 5:**
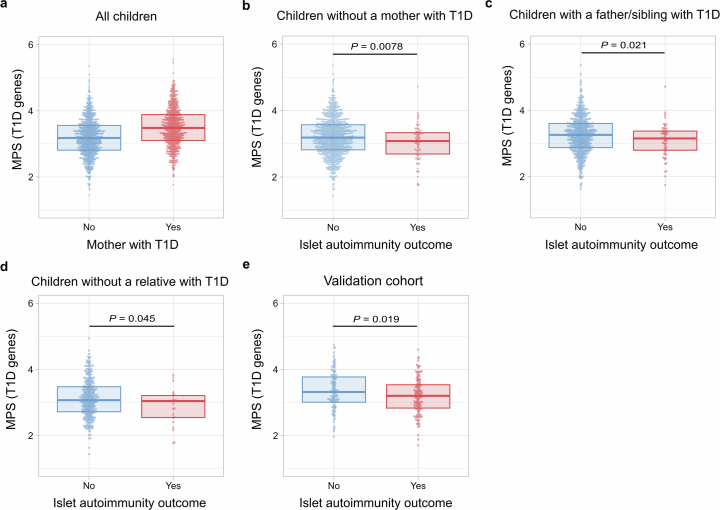
Methylation propensity score (MPS) of 28 CpGs linked to T1D susceptibility genes. **a**, The MPS based on 28 CpGs linked to T1D susceptibility genes by maternal T1D status (*n*_*total*_ = 1,752, *n* children with a mother with T1D = 790). **b**, The MPS based on 28 CpGs linked to T1D susceptibility genes in children not exposed to maternal T1D by islet autoimmunity outcome (*n*_*total*_ = 962, *n* children who developed islet autoimmunity = 81, adjusted logistic regression, two-sided). **c**, The MPS based on 28 CpGs linked to T1D susceptibility genes in children with a father and/or sibling with T1D by islet autoimmunity outcome (*n*_*total*_ = 603, *n* children who developed islet autoimmunity = 57, adjusted logistic regression, two-sided). **d**, The MPS based on 28 CpGs linked to T1D susceptibility genes in children without a first-degree relative with T1D by islet autoimmunity outcome (*n*_*total*_ = 359, *n* children who developed islet autoimmunity = 24, adjusted logistic regression, two-sided). **e**, Validation of the MPS based on 28 CpGs linked to T1D susceptibility in children without a mother with T1D from an independent cohort (Fr1da) by islet autoimmunity outcome (*n*_*total*_ = 244, *n* children who developed islet autoimmunity = 133, adjusted logistic regression, two-sided). Box-plots indicate the median and the 25^th^ and 75^th^ percentile of the individual samples (dots). Uncorrected *P*-values (two-sided), derived from logistic regression adjusted for sex and age, are presented. Source data

The association of the scores with islet autoimmunity risk was assessed in the offspring of mothers without T1D. We first examined the MPS for the T1D susceptible loci CpG set. The MPS was significantly lower in the offspring who later developed islet autoimmunity compared with offspring who did not develop islet autoimmunity (adjusted logistic regression; *P* = 0.0078; Fig. 5d). Each 1-unit increase in the MPS decreased the odds of islet autoimmunity by 43% (odds ratio (OR) (95% confidence interval (CI)): 0.57 (0.37‒0.86); *P* = 0.0078; Fig. 5e). Similar results were obtained when the offspring were stratified into those with a non-maternal affected family member (Fig. 5f) or those without affected family members (Fig. 5g). We validated this finding in an independent cohort (Fr1da study) of children with and without islet autoantibodies who did not have a mother with T1D (median age 4.8 years, IQR 3.4‒5.8 years). Children with islet autoimmunity had a significantly lower MPS for the T1D susceptible loci CpG set than children without islet autoimmunity (adjusted logistic regression; *P* = 0.014; Fig. 5h). A MPS using only the 28 CpGs linked to T1D susceptibility genes (Supplementary Table 6) showed similar significant differences between these children (Extended Data Fig. 5). In contrast to the associations with islet autoimmunity observed for the MPS for the T1D susceptible loci CpG set, we found no association with islet autoimmunity risk for the MPS for the non-susceptible loci CpG set (Fig. 5i).

In summary, the differentially methylated CpGs in offspring of mothers with T1D that potentially regulate T1D susceptibility were also associated with a reduced risk of islet autoimmunity in the offspring of unaffected mothers, suggesting that maternal T1D protects against islet autoimmunity via the epigenetic modification of T1D susceptibility loci.

## Discussion

Islet autoimmunity and T1D have a strong polygenic basis with a significant contribution from genes within the MHC HLA region on chromosome 6p21 (refs. ^11,12^). In this study, we show that exposure to maternal T1D, a major environmental factor that provides protection against islet autoimmunity in children compared with children with a father or sibling with T1D, is associated with extensive changes in blood CpG methylation across the offspring’s genome, 18 months or more after exposure. Many of these methylation changes were linked to immune-related genes and to genes conferring T1D susceptibility, particularly within the MHC region. Furthermore, using scores to capture the differences in CpG methylation observed in the offspring of mothers with T1D, we show that maternal T1D offspring methylation profiles for CpGs linked to T1D susceptibility genes are associated with protection against islet autoimmunity in the offspring of mothers without T1D. These findings indicate that the risk for islet autoimmunity can be modulated by environmental factors through the epigenetic modification of T1D susceptibility genes.

The T1D susceptible genetic load of offspring of mothers with T1D is similar to that of offspring whose fathers have T1D and whose mothers do not have diabetes. Despite this, the risk in offspring of mothers with T1D is about half that of the offspring of fathers with T1D^13^. This protective effect extends to islet autoimmunity developing in the first years of life but does not extend to other autoimmunity associated with T1D, for example, coeliac disease. This divergence in risk offers an opportunity to elucidate the mechanisms of protection against the development of islet autoimmunity and T1D. Previous studies in humans have revealed immunological differences in the offspring of mothers with T1D, including a relative scarcity of CD4^+^ T cells responsive to islet autoantigens at birth, compared with the offspring of mothers without T1D^16^. Our findings corroborate that protection is exerted through immune pathways. Among the 1,677 differentially methylated CpG observed in our study, many were linked to genes involved in immune pathways relevant to autoimmunity and T1D pathogenesis. The notable involvement of immune pathways includes the identification of motif enrichment for binding to the transcription corepressor MBD2. MBD2 is part of the nucleosome remodelling and histone deacetylation complex and is one of several proteins that bind to hypermethylated regions to modulate transcription. Consistent with this, most of the differentially methylated CpGs found in the offspring of mothers with T1D were hypermethylations. Current knowledge of the role of MBD2 in immunity is derived mainly from studies in *mbd2*-deficient mice. These mice exhibit impaired CD8^+^ effector and memory T cell maturation after viral infection^26^. Notably, mdb2 regulates the transcription of Forkhead-box-protein P3 (Foxp3), the master transcriptional regulator of regulatory T cells^28^. MBD2 promotes Tet methylcytosine dioxygenase 2 (TET2)-mediated demethylation of the T_reg_-specific demethylation region in T_reg_ cells, and *mbd2*-deficient mice exhibit impaired T_reg_ cell number and function^28^. On a non-obese diabetic background, *mbd2* deficiency upregulates T helper cell 1 programs and exacerbates the development of autoimmune diabetes, and ectopic mbd2 expression in CD4^+^ T cells reduces the development of diabetes in an adoptive transfer model^27^. Our findings, namely the methylation changes corresponding to increased MBD2 activity in the protective maternal T1D environment, are consistent with these experimental data.

The HOXA gene region in chromosome 7p15 was also enriched for CpGs that were differentially methylated in offspring of mothers with T1D. Beyond HOXA5, a major CpG target in our analysis with significant roles in chronic inflammation^22,23^, we discovered that HOX antisense intergenic RNA myeloid 1 (HOTAIRM1), a long non-coding RNA, was a particular target of the differentially methylated CpGs in the offspring of mothers with T1D, especially in the eQTM data. HOTAIRM1 is activated by Interferon regulatory factor 4 (IRF4) and in turn promotes *IRF4* expression in T cells^38^. IRF4 is a critical factor for the maturation of T and B cells, for the regulatory action of T_reg_s, and the differentiation of T helper 17 cells^39^. As a consequence, IRF4 has substantial effects on the immune cells that are relevant to the development of autoimmune diseases, such as T1D, and has been suggested as a promising therapeutic target^39^. Notably, differential methylation at 69 CpG sites targeting *HOTAIRM1* expression as indicated by eQTM data was observed in children of mothers with T1D. This epigenetic alteration was associated with reduced *HOTAIRM1* transcription, which may in turn lower IRF4 levels and contribute to a decreased risk of T1D. Functional studies using immune cell-specific *HOTAIRM1* knockdown in non-obese diabetic mice are warranted to investigate its impact on the development of autoimmunity.

Our study identified a large number of differentially methylated CpGs related to T1D susceptibility genes in the offspring of mothers with T1D, predominantly within the MHC region. Of the 15 T1D susceptibility genes, 11 were linked to the differentially methylated CpGs according to eQTM data. Most of these genes, however, have undefined roles in the development of T1D. According to the eQTM data, the differentially methylated CpGs in the offspring of mothers with T1D are predicted to lead to reduced gene expression of *SKAP2*. Higher levels of SKAP2 may contribute to the development of T1D via dysregulation of myeloid immune cells^40^. Other genes such as *Ring finger protein 5* (*RNF5*) and *SKI2 subunit of superkiller complex* (*SKIC2*) encode proteins that are involved in anti-viral responses^41,42^. Differential methylation at CpG sites close to the T1D susceptibility gene *TNXB* was also observed in monozygotic twins discordant for T1D^43^. Several CpGs targeted the expression of HLA Class I (*HLA-A* and *HLA-C*) and non-classical HLA genes (*HLA-F* and *HLA-G*). On the basis of the eQTM data, differential methylation in the offspring of mothers with T1D was predicted to increase *HLA-A* expression and decrease *HLA-C* expression. These opposing directions of expression are predictions. They may, however, reflect distinct roles of these genes in immune cell antigen recognition. HLA-A has been implicated with CD8⁺ T cell activation, whereas HLA-C is more closely associated with natural killer cell regulation^44^. A potential role of the non-classical HLA genes was suggested in the development of T1D on the basis of their key function of natural killer cell regulation^45^. HLA Class I also has a likely role in immune tolerance during pregnancy^46^. Our larger study in older children was, however, unable to validate our previous findings of differential methylation of CpG sites at the T1D susceptibility *INS* gene in cord blood^21^. Overall, our findings imply that exposure to maternal T1D can epigenetically modify and reduce the effect of genetic susceptibility to T1D in offspring. We suggest that the epigenetic remodelling of T1D susceptibility genes is likely to be common to other environmental modulators of T1D risk. These findings have potential therapeutic implications by way of epigenetic editing, DNA methyl transferase inhibitors, histone deacetylation inhibitors or mimics of the protective environment. For example, very early studies showed that neonatal exposure of diabetes-prone rats to high glucose could prevent diabetes development^17^.

We also show the potential for integrating epigenetic scores into current polygenic risk scores to predict islet autoimmunity and T1D. So far, no methylation score has been developed for T1D, although this approach has been evaluated for several other diseases, including allergy^47^ and type 2 diabetes^48^. We created the MPS to reflect methylation in offspring of mothers with T1D using only 34 CpGs linked to T1D susceptibility loci. This MPS was associated with protection against T1D in children who are not exposed to maternal T1D but who have an elevated genetic risk for T1D. This was observed in three separate cohorts. Therefore, although methylation may be unstable, methylation scores may enrich the predictability of genetic risk scores in the future. Furthermore, changes in methylation and, therefore, scores may also reflect alterations that affect risk as recently described^49^. Future evaluation of methylation sites and scores in various phases of T1D development, for example, after seroconversion and at different preclinical stages of T1D, with respect to risk prediction, progression to clinical T1D and therapeutic response to delay T1D onset, may allow a more precise risk assessment and the identification of individuals that benefit from therapeutic intervention. Protective modification of DNA methylation by therapeutics, lifestyle and environmental factors may provide opportunities to alter the risk of and progression to T1D.

A limitation of our study is that it was performed using whole-blood DNA, which did not allow us to determine the specific cellular subsets that are most affected by the methylation differences. As recommended for whole-blood analysis, we adjusted the analysis for six major blood cell types. There was no difference in the estimated frequency of these cell types between children with or without a mother with T1D, suggesting that the methylation differences did not result from a shift in individual cell types. Greater granularity could be achieved by assessing chromatin through techniques such as single cell assays for transposase-accessible chromatin sequencing or multi-omics. Differences were based on DNA methylation performed by array methods and confirmation by bisulfite pyrosequencing at a small number of CpGs. Our study was limited to public methylation-associated expression data from children in mid-childhood. We cannot exclude that the difference in age between our study and the children participating in the HELIX study influenced the associations between DNA methylation and gene expression. To our knowledge, the HELIX data are currently the only publicly available resource in childhood. Another limitation is that methylation was measured at a single time point. While an important finding was that the methylation differences were present at least 18 months after exposure, we cannot assess whether CpG methylation diverged further between children with and without a mother with T1D at an earlier age or whether the differences persist or become diluted by other environmental epigenetic modifiers with age. Furthermore, it is also possible that some of the differential methylation in the offspring are due to intergenerational transfer of epigenetic information differences between mothers and fathers with T1D as recently shown for obesity^50^. We are unable to assess this with our data. Our study was also limited to children who were mostly of European descent. Further studies should assess the epigenetic changes in single immune cells across multiple time points in individuals born to mothers with T1D with diverse ancestry backgrounds and in relation to intergenerational information transfer from mothers and fathers with T1D.

In summary, this analysis of differences in CpG methylation and in methylation regions between offspring of mothers with and without T1D suggests that this environmental modification of T1D risk is mediated by epigenetic changes at disease susceptibility genes. Further studies should address the therapeutic opportunities of these epigenetic changes.

## Methods

### Ethical statements

The BABYDIAB and BABYDIET studies were approved by the ethics committee of Bavaria, Germany (Bayerische Landesärztekammer no. 95357 and Ludwig-Maximilians University no. 329/00, respectively). Written informed consent to participate in the study was obtained from all study participants or their legal representatives. None of the participants were compensated.

POInT was approved by the institutional review boards and regulatory authorities of the Technische Universität München, Medical Faculty (326/17 Af), the Medical University of Warsaw (199/2017), the UK Health Research Authority (18/SC/0019), Onderzoek UZ/KU Leuven (S60711) and Regionala Etikprövningsnämnden i Lund (2017/918). Written informed consent to participate in the study was obtained from legal representatives of all study participants. A separate informed consent from the participating families was required to allow biobank storage of material, such as the DNA used in this study. None of the participants were compensated.

The Fr1da study was approved by the institutional review board at the Technical University of Munich (70/14). Written, informed consent was obtained from the children’s parents or legal guardians. None of the participants were compensated.

### Cohorts

#### BABYDIAB/BABYDIET

BABYDIAB and BABYDIET are prospective birth cohorts of individuals with a first-degree family history of T1D and who were born in Germany between 1989 and 2006^18,19^. The primary aim of these studies was to investigate the natural history of islet autoimmunity and T1D. Blood DNA samples were collected at the age of 2 years or at 1 of the next follow-up visits if not collected at age 2 years. We included 958 children in the present analysis for whom consent was given to use their DNA for genetic research and whose DNA was of adequate quality and quantity for EWAS analysis. Among these, 608 children had a mother with T1D and 350 children had a father and/or sibling with T1D and were born to a mother without diabetes (Extended Data Fig. 1a).

#### POInT

POInT is a randomized, controlled and multicentre clinical trial organized through the Global Platform for the Prevention of Autoimmune Diabetes (GPPAD)^20^. It is investigating whether daily intake of oral insulin reduces the incidence of islet autoimmunity and/or T1D in children at increased risk of T1D (ClinicalTrials.gov registration no. NCT03364868). Between 2018 and 2021 a total of 1,050 infants were enrolled at 4.0‒7.0 months of age. Infants were eligible if they had a predicted genetic risk of >10% for developing multiple islet autoantibodies by the age of 6.0 years. Blood DNA samples were collected at the age of 1.5 years. We included 794 children in the present analysis for whom consent was given to use their DNA for genetic research, and whose DNA was of adequate quality and quantity for EWAS analysis. Of these, 182 had a mother with T1D, 253 had a father and/or sibling with T1D and 359 had no first-degree relative with T1D (Extended Data Fig. 1b).

#### Fr1da

The Fr1da study is an ongoing public health screening programme for islet autoantibodies in Bavaria, Germany^51^. Between February 2015 and March 2024, 194,696 children aged 1.75‒10.99 years were screened for islet autoantibodies by primary care paediatricians during routine medical check-ups. We used whole-blood samples from 133 children who were identified with 2 or more persistent confirmed islet autoantibodies and of 111 age-matched and sex-matched control children who were islet autoantibody negative at screening (Extended Data Fig. 1c and Supplementary Table 1). All children included in the present analysis had mothers without T1D. For all children, consent was given to use their DNA for genetic research and DNA of adequate quality and quantity was available for methylation analysis.

### DNA methylation measurement

After extraction, genomic DNA was purified and 750 ng of bisulfite converted using the EZ-96-DNA methylation kit (cat. no. D5008, Zymo Research). We performed a genome-wide DNA methylation analysis using the Infinium MethylationEPIC (850K) Bead-Chip array (Illumina). The methylation measurements were carried out at Helmholtz Center Munich for samples from the BABYDIAB/BABYDIET studies and at Life & Brain for samples from the POInT and Fr1da studies. Samples were randomized across the chips by age and sex. The Bead-Chips were imaged using an Illumina iScan and the raw fluorescence intensities of the images were extracted using the GenomeStudio Methylation module (Illumina).

Preprocessing and quality control of the BABYDIAB/BABYDIET, POInT and Fr1da studies were identically performed using R software (v.4.3.2) with the R package ENmix. The methylation probes from downstream analysis were excluded if their detection *P* value was >10^−6^ and if the bead count was fewer than 3 in at least 5% of the sample. Samples where <99% of the probes had a detection *P* value of <10^−6^ and samples that had a sex mismatch were also excluded. Data were normalized using quantile normalization as implemented in ENmix. Last, we excluded probes on sex chromosomes, cross-hybridizing probes and probes located near single nucleotide polymorphisms^52,53^.

### Epigenetic age calculation

We estimated the methylation age of the children according to Horvath^54^ using the methylclock R package. Among various methods to calculate epigenetic age, Horvath’s method has been shown to be the most accurate for children’s blood DNA methylation^55^.

### Meta-analysis of epigenome-wide associations with maternal T1D

Associations between the maternal T1D status of the offspring (no or yes) and the children’s blood DNA methylation were analysed by robust linear regression using the R package limma. Methylation *M* values (logit-transformed *β* values) were used for all analyses. To adjust for potential technical effects, the first three principal components, covering over 95% of the variation, were included (Extended Data Fig. 1d). The blood cell compositions of six blood cell types (CD4^+^ T cells, CD8^+^ T cells, B cells, NK cells, neutrophils and monocytes) were estimated from a reference panel using the R package FlowSorted.Blood.EPIC. Regression models were adjusted for age at DNA sample, sex, the first three principal components and the six estimated blood cell types. Extreme methylation outliers were excluded on the basis of the Tukey method, as previously described^56^. Before the meta-analysis, the quality of the EWAS results was checked using the R package QCEWAS. Correction for bias and inflation was performed using the R package bacon. A fixed-effects invariance-weighted meta-analysis using the 686,621 probes common to all studies was conducted using the R package metafor. The meta-analysis showed good model estimates (inflation *λ* = 1.04, bias *µ* = 0.07). We excluded 35,350 CpGs with high heterogeneity between the studies (*I*^2^ > 75), leaving a total of 651,271 CpGs for the downstream analysis.

In addition to the individual CpG analysis, the DMRs were examined using the R package dmrff, applying the same models as described above. Among the various tools available for DMR analysis, dmrff has been shown to overcome the limitations of other R packages for DMR analysis and to efficiently control the false-positive rates^57^. The analysis of DMR was performed using the dmr.meta function with default settings (maximal distance between consecutive CpGs = 500 base pairs) in the dmrff package.

Methylation sites were annotated to the human genome (reference genome GRCh37/hg19) using the R package IlluminaHumanMethylationEPICanno.ilm10b4.hg19. The false-discovery rate (FDR) according to Benjamini–Hochberg was used to correct for multiple testing if not specified otherwise, and *P*_FDR_ < 0.05 (two-sided) was considered statistically significant.

### Validation of CpG methylation by bisulfite pyrosequencing

We performed target-specific bisulfite pyrosequencing on a subset of up to 74 children with or without a mother with T1D matched for age (median 2.08 years, IQR 2.0‒2.2 years) and sex. Target CpGs for validation at *HOXA5* and eQTM-confirmed T1D susceptibility genes were selected on the basis of significant methylation differences (nominal *P* < 0.05) at the array between the analysed children with or without a mother with T1D. One microgram of whole-blood DNA was bisulfite converted using the EZ-96-DNA Methylation Gold kit (cat. no. D5008, Zymo Research). Bisulfite-treated DNA was amplified by target-specific PCR using the HotStarTaq kit (cat. no. 203443, Qiagen) or ZymoTaq Premix (cat. no. E2003, Zymo Research) and the following PCR steps: polymerase activation at 95 °C for 15 min (10 min for ZymoTaq), 45 cycles of denaturation at 94 °C for 30 s, annealing at a variable temperature (below) for 45 s, elongation at 72 °C for 1 min and final elongation at 72 °C for 10 min (7 min for ZymoTaq). Target-specific PCR primers were obtained from Qiagen or designed using the PyroMark Assay Design software v.2.0.1.15. The following primer assays were obtained from Qiagen to target CpGs at *HOXA5* (cat. no. 978776, GeneGlobeID: Hs_HOXA5_03_PM, targeting cg04863892, PCR annealing temperature 50 °C; GeneGlobeID: Hs_HOXA5_09_PM, targeting cg17432857, cg00969405, PCR annealing temperature 54 °C). The following primers (original sequence 5′ to 3′) were designed to target CpGs at T1D susceptibility genes (cg00106345 at *SKAP2*: forward primer TTGGCCCTCTAGGCAAGTAGGTCAG, biotinylated reverse primer GAGGCCCTGAACTGTTCATGGCAT, PCR annealing temperature 52 °C, sequencing primer GGGAGACTGGGTGAA; cg27003765 and cg07357081 at *SLC44A4*/*LY6G5B*: forward primer GCCCGCTGGGCACAAAGTTGAGAAGAAGGA, biotinylated reverse primer GAACTAAGGAGAGTACTGTGTCCCTGAGGG, PCR annealing temperature 56 °C, sequencing primer CCAGGTCTCCAGGGCTCCAA; cg26266427 and cg01337207 at *TNXB*/*SKIC2*, forward primer AGGATTGCGGTGTGAGGCAGTG, biotinylated reverse primer AGAGCCTCCAGCCAGCGCCTGCCCTGGAGG, PCR annealing temperature 52 °C, sequencing primer AGTGCCCGAATGACTGCAGCCAGCA). Designed primers were obtained from Integrated DNA Technologies. All primers were checked for amplicon specificity using the in silico PCR function provided by the UCSC Genome Browser and by agarose gel electrophoresis. Pyrosequencing on the amplified PCR products was performed on a PyroMark Q24 instrument (Qiagen) using the PyroMark Q24 Gold reagents (cat. no. 970802, Qiagen). Analysis was done using the PyroMark Q24 Analysis software v.2.0.7 (Qiagen). Only pyrosequencing runs that passed the integrated quality assessment were included in the analysis. Because of the high input DNA amount per assay, some assays could not be performed in the whole subset. Methylation levels between the groups were statistically compared using Welch’s *t*-test or Mann–Whitney *U*-test, depending on normal distribution of the data, and unadjusted *P* values are given (two-sided).

### Heatmaps of CpGs associated with maternal T1D

Methylation *M* values were adjusted for the same covariables as in the EWAS model, *z*-scored and plotted by children with a mother with T1D and children without an affected mother. Heatmaps were created using the R package pheatmap using the Euclidean distance method to cluster the CpGs.

### Association analysis between variables and CpG methylation

The potential influence of early-life (maternal-related and pregnancy-related) factors on the observed DNA methylation differences in children born to mothers with T1D was assessed using adjusted linear regression between CpG methylation and maternal age at delivery, maternal HbA1c in the last trimester of pregnancy, parity, delivery by Caesarean section and birth weight. Models were adjusted for age at DNA sample, sex, the first three principal components and the six estimated blood cell types. The maternal-related and pregnancy-related variables were analysed in the BABYDIAB/BABYDIET study.

### Enrichment analysis of locations and transcription factor motifs

All CpGs included in the analyses were annotated to their genetic region using the R package annotatr and hg19 as the reference genome. Hypergeometric tests were used to determine region enrichment or depletion on the basis of the CpGs per genetic region among the identified CpGs and all CpGs included in the meta-analyses using the phyper function implemented in R. Annotations to CpG island classifications were extracted from the Illumina annotation file. The *P* values were adjusted for the six region types or island classifications using Bonferroni correction. The Roadmap ChromHMM marks of primary blood cells from peripheral blood and T1D-relevant tissues^58^ were used to perform enrichment analysis for regulatory elements. The *P* values were adjusted for the six regulatory element types using Bonferroni correction. Motif enrichment analysis of the transcription factor binding sites was performed using the ELMER package in R using all differentially methylated CpGs found in the offspring of mothers with T1D. Only the motifs of class A and B (strong confidence) were examined. Motifs with an OR > 1 and *P*_FDR_ < 0.05 were considered significantly enriched.

### Functional evaluation of CpGs

To assess whether the identified methylation sites were associated with blood-specific gene expression, we queried the HELIX *cis*-eQTM catalogue (adjusted for blood cell types) of blood from 832 children with a mean age of 8.1 years (Illumina 450 K methylation array only)^59^. The EWAS catalogue was queried for protein traits associated with the identified CpGs^60^.

### Gene ontology analysis

Gene ontology analysis was performed using Panther^61^. Over-representation of ontologies among the input genes was assessed using Fisher’s exact test (*P*_FDR_ < 0.05).

### Identification of genes, proteins and methylation loci

To determine whether the CpG gene targets were previously identified as T1D susceptibility genes, we queried the Harmonizome database for T1D, which included 144 distinct genes associated with T1D^62^ and the Genome-Wide Association Studies catalogue, which included 335 genes. For proteins related to T1D development, we searched PubMed using the terms ‘protein biomarkers’ AND ‘type 1 diabetes’ (search included publications until 30 May 2024) and compared the identified biomarker proteins in matching studies^35,63–67^ with the associated protein traits. Enrichment among the associated CpGs was determined using a hypergeometric test with Bonferroni correction for multiple testing (*P* < 1.64 × 10^−4^). Furthermore, we assessed the overlap between maternal T1D-associated CpGs and DMRs and methylation loci in blood previously linked to T1D development and patients with T1D^43,68–71^.

### Development of the MPS for offspring of mothers with T1D

We generated MPSs for the offspring of mothers with T1D to capture their methylation differences. The CpGs were first stratified into those associated with T1D susceptibility loci and the remainder. For CpGs strongly correlated with each other (Pearson’s *r* > 0.8), we randomly excluded one correlated CpG. Random-forest recursive feature elimination was applied using the R package caret to identify the CpGs with the highest importance for predicting offspring of a mother with T1D. We split the data by study into training (BABYDIAB/BABYDIET) and test (POInT) sets. The recommended models were then used to generate the MPSs by a weighted sum of the individual methylation levels using the R package tidymodels. We performed logistic regression with age at DNA methylation and sex to assess the association of the MPSs with the islet autoimmunity outcome, that is, persistent confirmed multiple islet antibodies and/or T1D, in the offspring of mothers without T1D. The association between the MPSs and the islet autoantibody outcome was validated using an independent cohort of 244 children from the Fr1da study (Supplementary Table 1). A *P* value <0.05 (two-sided) was considered statistically significant.

### Statistical analysis

Data collection and analysis were not performed blind to the conditions of the experiments. No samples were excluded after quality control of the methylation data (Extended Data Fig. 1a,b). Extreme methylation values, as defined by the Tukey method, were excluded before the EWAS. In addition, methylation sites showing high heterogeneity (*I*^2^ > 75) between the meta-analysed studies were excluded, as described above. We used R software (v.4.3.2) for all statistical analysis and graphical illustrations. Data met the assumptions of the respective statistical tests used. Normality was tested using Shapiro–Wilk test and the *F*-test applied to check for equal variances. Data were not transformed to achieve normal distribution. Respective statistical tests and multiple testing correction methods are reported within Methods. *P* values were two-sided, except for enrichment analysis (one-sided).

### Figure alignment

Inkscape software (v.1.0.2) was used for schematic illustrations and figure alignment.

### Reporting summary

Further information on research design is available in the Nature Portfolio Reporting Summary linked to this article.

## Supplementary information

Reporting Summary

Supplementary Tables 1–6 Supplementary Table 1. Characteristics of the included children participating in the BABYDIAB/BABYDIET, POInT and Fr1da studies. Supplementary Table 2. Differential methylated CpGs and DMRs in offspring with maternal T1D. Supplementary Table 3. Overlap between maternal T1D-associated CpGs and DMRs and blood eQTM in childhood. Supplementary Table 4. Identified CpGs associated with circulating proteins in the EWAS catalogue that showed significant relations to progression or onset of T1D. Supplementary Table 5. Overlap of CpGs and DMRs with previously published studies on T1D. Supplementary Table 6. CpGs used for the MPS on maternal T1D.

## Source data

Source Data Fig. 1 Statistical source data.

Source Data Fig. 2 Statistical source data.

Source Data Fig. 3 Statistical source data.

Source Data Fig. 4 Statistical source data.

Source Data Fig. 5 Statistical source data.

Source Data Extended Data Fig. 1 Statistical source data.

Source Data Extended Data Fig. 2 Statistical source data.

Source Data Extended Data Fig. 3 Statistical source data.

Source Data Extended Data Fig. 4 Statistical source data.

Source Data Extended Data Fig. 5 Statistical source data.

## Data Availability

We have included comprehensive summary statistics of all significant results within the main text and Supplementary Tables 1–6. The publicly available data used in this study can be accessed from the HELIX project at https://helixomics.isglobal.org/downloads/downloads.html and the EWAS catalogue repositories at https://www.ewascatalog.org/download/. Full summary statistics of the meta-EWAS are available via Zenodo at 10.5281/zenodo.17018103 (ref. ^72^). The data that support the findings of this study are available from the corresponding author upon reasonable request. Source data are provided with this paper.

## Code availability

All software used is publicly available and described in Methods. Preprocessing and normalization of methylation data was performed using the R package ENmix (v.1.40.2). Blood cells were estimated using the R package FlowSorted.Blood.EPIC (v.2.8.0). Epigenome-wide association analysis was performed using the R package limma (v.3.50.0). Quality control of the EWAS results was checked using the R package QCEWAS (v.1.2.3). Epigenetic age was calculated using the R package methylclock (v.1.14.0). The meta-analysis was performed using the R package metafor (v.3.0-2) with correction for bias and inflation using the R package bacon (v.1.30.0). DMR analysis was carried out with the R package dmrff (v.1.0.0) using the function dmr.meta. Heatmaps were created with the R package pheatmap (v.1.10.12). Hypergeometric tests were performed using the phyper function of the R package stats (v.4.3.2). Motif enrichment analysis was done using the R package ELMER (v.2.26.0). Recursive feature elimination was performed using the rfe function in the R package caret (v.6.0-92) and models for the MPS were created with the R package tidymodels (v.1.2.0). Customized code for the EWAS analysis, generation of the MPS and the figures is publicly available via GitHub at https://github.com/rrepi/mt1d/.

## References

[1] Gillman, M. W. Developmental origins of health and disease. *N. Engl. J. Med.* **353**, 1848–1850 (2005). 16251542 10.1056/NEJMe058187 PMC1488726

[2] Cortessis, V. K. et al. Environmental epigenetics: prospects for studying epigenetic mediation of exposure-response relationships. *Hum. Genet* **131**, 1565–1589 (2012). 22740325 10.1007/s00439-012-1189-8 PMC3432200

[3] Tobi, E. W. et al. DNA methylation signatures link prenatal famine exposure to growth and metabolism. *Nat. Commun.* **5**, 5592 (2014). 25424739 10.1038/ncomms6592 PMC4246417

[4] Maitre, L. et al. Multi-omics signatures of the human early life exposome. *Nat. Commun.* **13**, 7024 (2022). 36411288 10.1038/s41467-022-34422-2 PMC9678903

[5] Joubert, B. R. et al. DNA methylation in newborns and maternal smoking in pregnancy: genome-wide consortium meta-analysis. *Am. J. Hum. Genet* **98**, 680–696 (2016). 27040690 10.1016/j.ajhg.2016.02.019 PMC4833289

[6] Kotsakis Ruehlmann, A., et al. Epigenome-wide meta-analysis of prenatal maternal stressful life events and newborn DNA methylation. *Mol. Psychiatry*10.1038/s41380-023-02010-5 (2023). 36899042 10.1038/s41380-023-02010-5 PMC12486153

[7] Sharp, G. C. et al. Maternal BMI at the start of pregnancy and offspring epigenome-wide DNA methylation: findings from the pregnancy and childhood epigenetics (PACE) consortium. *Hum. Mol. Genet* **26**, 4067–4085 (2017). 29016858 10.1093/hmg/ddx290 PMC5656174

[8] Dominguez-Salas, P. et al. Maternal nutrition at conception modulates DNA methylation of human metastable epialleles. *Nat. Commun.* **5**, 3746 (2014). 24781383 10.1038/ncomms4746 PMC4015319

[9] Ward, Z. J. et al. Estimating the total incidence of type 1 diabetes in children and adolescents aged 0-19 years from 1990 to 2050: a global simulation-based analysis. *Lancet Diabetes Endocrinol.* **10**, 848–858 (2022). 36372070 10.1016/S2213-8587(22)00276-5

[10] Ziegler, A.-G. The countdown to type 1 diabetes: when, how and why does the clock start?. *Diabetologia* **66**, 1169–1178 (2023). 37231274 10.1007/s00125-023-05927-2 PMC10212739

[11] Bluestone, J. A., Herold, K. & Eisenbarth, G. Genetics, pathogenesis and clinical interventions in type 1 diabetes. *Nature* **464**, 1293–1300 (2010). 20432533 10.1038/nature08933 PMC4959889

[12] Todd, J. A. Etiology of type 1 diabetes. *Immunity* **32**, 457–467 (2010). 20412756 10.1016/j.immuni.2010.04.001

[13] Rewers, M., Stene, L. C. & Norris, J. M. in *Diabetes in America* 3rd edn (eds Rewers, M. et al.) Chap. 11 (National Institute of Diabetes and Digestive and Kidney Diseases, 2018).

[14] Bonifacio, E. et al. Maternal type 1 diabetes reduces the risk of islet autoantibodies: relationships with birthweight and maternal HbA(1c). *Diabetologia* **51**, 1245–1252 (2008). 18463843 10.1007/s00125-008-1022-z

[15] Koczwara, K., Bonifacio, E. & Ziegler, A. G. Transmission of maternal islet antibodies and risk of autoimmune diabetes in offspring of mothers with type 1 diabetes. *Diabetes* **53**, 1–4 (2004). 14693690 10.2337/diabetes.53.1.1

[16] Knoop, J. et al. Maternal type 1 diabetes reduces autoantigen-responsive CD4(+) T cells in offspring. *Diabetes* **69**, 661–669 (2020). 31896551 10.2337/db19-0751

[17] Buschard, K., Jørgensen, M., Aaen, K., Bock, T. & Josefsen, K. Prevention of diabetes mellitus in BB rats by neonatal stimulation of beta cells. *Lancet* **335**, 134–135 (1990). 1967433 10.1016/0140-6736(90)90004-o

[18] Ziegler, A. G., Hummel, M., Schenker, M. & Bonifacio, E. Autoantibody appearance and risk for development of childhood diabetes in offspring of parents with type 1 diabetes: the 2-year analysis of the German BABYDIAB Study. *Diabetes* **48**, 460–468 (1999). 10078544 10.2337/diabetes.48.3.460

[19] Hummel, S., Pflüger, M., Hummel, M., Bonifacio, E. & Ziegler, A. G. Primary dietary intervention study to reduce the risk of islet autoimmunity in children at increased risk for type 1 diabetes: the BABYDIET study. *Diabetes Care* **34**, 1301–1305 (2011). 21515839 10.2337/dc10-2456 PMC3114350

[20] Ziegler, A. G. et al. Oral insulin therapy for primary prevention of type 1 diabetes in infants with high genetic risk: the GPPAD-POInT (Global Platform for the Prevention of Autoimmune Diabetes Primary Oral Insulin Trial) study protocol. *BMJ Open* **9**, e028578 (2019). 31256036 10.1136/bmjopen-2018-028578 PMC6609035

[21] Hubert, K. A. & Wellik, D. M. Hox genes in development and beyond. *Development*10.1242/dev.192476 (2023). 10.1242/dev.192476 PMC10216783 36645372

[22] Parrillo, L. et al. The transcription factor HOXA5: novel insights into metabolic diseases and adipose tissue dysfunction. *Cells*10.3390/cells12162090 (2023). 10.3390/cells12162090 PMC10453582 37626900

[23] Cao, W. et al. Hoxa5 alleviates obesity-induced chronic inflammation by reducing ER stress and promoting M2 macrophage polarization in mouse adipose tissue. *J. Cell. Mol. Med.* **23**, 7029–7042 (2019). 31441588 10.1111/jcmm.14600 PMC6787506

[24] Knorr, S. et al. Epigenetic and transcriptomic alterations in offspring born to women with type 1 diabetes (the EPICOM study). *BMC Med* **20**, 338 (2022). 36138412 10.1186/s12916-022-02514-x PMC9503228

[25] Cheng, L. et al. Loss of MBD2 affects early T cell development by inhibiting the WNT signaling pathway. *Exp. Cell. Res.* **398**, 112400 (2021). 33271126 10.1016/j.yexcr.2020.112400

[26] Kersh, E. N. Impaired memory CD8 T cell development in the absence of methyl-CpG-binding domain protein 2. *J. Immunol.* **177**, 3821–3826 (2006). 16951344 10.4049/jimmunol.177.6.3821

[27] Yue, T. et al. MBD2 acts as a repressor to maintain the homeostasis of the Th1 program in type 1 diabetes by regulating the STAT1-IFN-γ axis. *Cell Death Differ.* **29**, 218–229 (2022). 34420035 10.1038/s41418-021-00852-6 PMC8738722

[28] Wang, L. et al. Mbd2 promotes foxp3 demethylation and T-regulatory-cell function. *Mol. Cell. Biol.* **33**, 4106–4115 (2013). 23979593 10.1128/MCB.00144-13 PMC3811679

[29] Roep, B. O. The role of T-cells in the pathogenesis of Type 1 diabetes: from cause to cure. *Diabetologia* **46**, 305–321 (2003). 12687328 10.1007/s00125-003-1089-5

[30] Gearty, S. V. et al. An autoimmune stem-like CD8 T cell population drives type 1 diabetes. *Nature* **602**, 156–161 (2022). 34847567 10.1038/s41586-021-04248-x PMC9315050

[31] Nejentsev, S. et al. Localization of type 1 diabetes susceptibility to the MHC class I genes HLA-B and HLA-A. *Nature* **450**, 887–892 (2007). 18004301 10.1038/nature06406 PMC2703779

[32] Onengut-Gumuscu, S. et al. Fine mapping of type 1 diabetes susceptibility loci and evidence for colocalization of causal variants with lymphoid gene enhancers. *Nat. Genet.* **47**, 381–386 (2015). 25751624 10.1038/ng.3245 PMC4380767

[33] James, I., McKinnon, E., Gaudieri, S. & Morahan, G. Missingness in the T1DGC MHC fine-mapping SNP data: association with HLA genotype and potential influence on genetic association studies. *Diabetes Obes. Metab.* **11**, 101–107 (2009). 19143822 10.1111/j.1463-1326.2008.01010.x PMC2755067

[34] Sticht, J., Álvaro-Benito, M. & Konigorski, S. Type 1 diabetes and the HLA region: genetic association besides classical HLA Class II genes. *Front. Genet.* **12**, 683946 (2021). 34220961 10.3389/fgene.2021.683946 PMC8248358

[35] Nakayasu, E. S. et al. Plasma protein biomarkers predict the development of persistent autoantibodies and type 1 diabetes 6 months prior to the onset of autoimmunity. *Cell Rep. Med* **4**, 101093 (2023). 37390828 10.1016/j.xcrm.2023.101093 PMC10394168

[36] Jailwala, P. et al. Apoptosis of CD4^+^ CD25(high) T cells in type 1 diabetes may be partially mediated by IL-2 deprivation. *PLoS ONE* **4**, e6527 (2009). 19654878 10.1371/journal.pone.0006527 PMC2716541

[37] Hosseini, M., Lotfi-Shahreza, M. & Nikpour, P. Integrative analysis of DNA methylation and gene expression through machine learning identifies stomach cancer diagnostic and prognostic biomarkers. *J. Cell. Mol. Med.* **27**, 714–726 (2023). 36779430 10.1111/jcmm.17693 PMC9983314

[38] Li, L. et al. IRF4 transcriptionally activate HOTAIRM1, which in turn regulates IRF4 expression, thereby affecting Th9 cell differentiation and involved in allergic rhinitis. *Gene* **813**, 146118 (2022). 34929342 10.1016/j.gene.2021.146118

[39] Xu, W.-D., Pan, H.-F., Ye, D.-Q. & Xu, Y. Targeting IRF4 in autoimmune diseases. *Autoimmun. Rev.* **11**, 918–924 (2012). 23010632 10.1016/j.autrev.2012.08.011

[40] Rutsch, N. et al. Diabetes with multiple autoimmune and inflammatory conditions linked to an activating SKAP2 mutation. *Diabetes Care* **44**, 1816–1825 (2021). 34172489 10.2337/dc20-2317 PMC8385470

[41] Ge, J. & Zhang, L. RNF5: inhibiting antiviral immunity and shaping virus life cycle. *Front. Immunol.* **14**, 1324516 (2023). 38250078 10.3389/fimmu.2023.1324516 PMC10796512

[42] Aly, H. H. et al. RNA exosome complex regulates stability of the hepatitis B virus X-mRNA transcript in a non-stop-mediated (NSD) RNA quality control mechanism. *J. Biol. Chem.* **291**, 15958–15974 (2016). 27281821 10.1074/jbc.M116.724641 PMC4965548

[43] Elboudwarej, E. et al. Hypomethylation within gene promoter regions and type 1 diabetes in discordant monozygotic twins. *J. Autoimmun.* **68**, 23–29 (2016). 26782299 10.1016/j.jaut.2015.12.003 PMC4792657

[44] Vollmers, S., Lobermeyer, A. & Körner, C. The new kid on the block: HLA-C, a key regulator of natural killer cells in viral immunity. *Cells*10.3390/cells10113108 (2021). 10.3390/cells10113108 PMC8620871 34831331

[45] Wyatt, R. C., Lanzoni, G., Russell, M. A., Gerling, I. & Richardson, S. J. What the HLA-I!–Classical and non-classical HLA Class I and their potential roles in type 1 diabetes. *Curr. Diab Rep.* **19**, 159 (2019). 31820163 10.1007/s11892-019-1245-z PMC6901423

[46] Persson, G., Jørgensen, N., Nilsson, L. L., Andersen, L. H. J. & Hviid, T. V. F. A role for both HLA-F and HLA-G in reproduction and during pregnancy?. *Hum. Immunol.* **81**, 127–133 (2020). 31558330 10.1016/j.humimm.2019.09.006

[47] Kilanowski, A. et al. Methylation risk scores for childhood aeroallergen sensitization: results from the LISA birth cohort. *Allergy* **77**, 2803–2817 (2022). 35437756 10.1111/all.15315 PMC9437118

[48] Cheng, Y. et al. Development and validation of DNA methylation scores in two European cohorts augment 10-year risk prediction of type 2 diabetes. *Nat. Aging* **3**, 450–458 (2023). 37117793 10.1038/s43587-023-00391-4

[49] Carry, P. M. et al. Longitudinal changes in DNA methylation during the onset of islet autoimmunity differentiate between reversion versus progression of islet autoimmunity. *Front. Immunol.*10.3389/fimmu.2024.1345494 (2024). 10.3389/fimmu.2024.1345494 PMC11194352 38915393

[50] Tomar, A. et al. Epigenetic inheritance of diet-induced and sperm-borne mitochondrial RNAs. *Nature* **630**, 720–727 (2024). 38839949 10.1038/s41586-024-07472-3 PMC11186758

[51] Ziegler, A. G. et al. Yield of a public health screening of children for islet autoantibodies in Bavaria, Germany. *JAMA* **323**, 339–351 (2020). 31990315 10.1001/jama.2019.21565 PMC6990943

[52] Pidsley, R. et al. Critical evaluation of the Illumina MethylationEPIC BeadChip microarray for whole-genome DNA methylation profiling. *Genome Biol.* **17**, 208 (2016). 27717381 10.1186/s13059-016-1066-1 PMC5055731

[53] Zhou, W., Laird, P. W. & Shen, H. Comprehensive characterization, annotation and innovative use of Infinium DNA methylation BeadChip probes. *Nucleic Acids Res.* **45**, e22 (2017). 27924034 10.1093/nar/gkw967 PMC5389466

[54] Horvath, S. DNA methylation age of human tissues and cell types. *Genome Biol.* **14**, R115 (2013). 24138928 10.1186/gb-2013-14-10-r115 PMC4015143

[55] Fang, F. et al. Evaluation of pediatric epigenetic clocks across multiple tissues. *Clin. Epigenetics* **15**, 142 (2023). 37660147 10.1186/s13148-023-01552-3 PMC10475199

[56] Ott, R. et al. Epigenome-wide meta-analysis reveals associations between dietary glycemic index and glycemic load and DNA methylation in children and adolescents of different body sizes. *Diabetes Care* **46**, 2067–2075 (2023). 37756535 10.2337/dc23-0474

[57] Suderman, M. et al. dmrff: identifying differentially methylated regions efficiently with power and control. Preprint at *bioRxiv*10.1101/508556 (2018).

[58] Kundaje, A. et al. Integrative analysis of 111 reference human epigenomes. *Nature* **518**, 317–330 (2015). 25693563 10.1038/nature14248 PMC4530010

[59] Ruiz-Arenas, C. et al. Identification of autosomal cis expression quantitative trait methylation (*cis*eQTMs) in children’s blood. *Elife*10.7554/eLife.65310 (2022). 10.7554/eLife.65310 PMC8933004 35302492

[60] Battram, T. et al. The EWAS Catalog: a database of epigenome-wide association studies. *Wellcome Open Res.* **7**, 41 (2022). 35592546 10.12688/wellcomeopenres.17598.1 PMC9096146

[61] Mi, H., Muruganujan, A. & Thomas, P. D. PANTHER in 2013: modeling the evolution of gene function, and other gene attributes, in the context of phylogenetic trees. *Nucleic Acids Res.* **41**, D377–D386 (2013). 23193289 10.1093/nar/gks1118 PMC3531194

[62] Rouillard, A. D. et al. The harmonizome: a collection of processed datasets gathered to serve and mine knowledge about genes and proteins. *Database* **2016**, baw100 (2016). 27374120 10.1093/database/baw100 PMC4930834

[63] Yazdanpanah, N. et al. Clinically relevant circulating protein biomarkers for type 1 diabetes: evidence from a two-sample Mendelian randomization study. *Diabetes Care* **45**, 169–177 (2022). 34758976 10.2337/dc21-1049

[64] Webb-Robertson, B. M. et al. Decrease in multiple complement proteins associated with development of islet autoimmunity and type 1 diabetes. *iScience* **27**, 108769 (2024). 38303689 10.1016/j.isci.2023.108769 PMC10831269

[65] Sarkar, S. et al. Systematic review of type 1 diabetes biomarkers reveals regulation in circulating proteins related to complement, lipid metabolism, and immune response. *Clin. Proteom.* **20**, 38 (2023). 10.1186/s12014-023-09429-6 PMC10512508 37735622

[66] Metz, T. O. et al. Application of proteomics in the discovery of candidate protein biomarkers in a diabetes autoantibody standardization program sample subset. *J. Proteome Res* **7**, 698–707 (2008). 18092746 10.1021/pr700606w PMC2672959

[67] Nogueira, V. C. et al. UPLC-HDMS(E) to discover serum biomarkers in adults with type 1 diabetes. *Int. J. Biol. Macromol.* **221**, 1161–1170 (2022). 36115450 10.1016/j.ijbiomac.2022.09.085

[68] Johnson, R. K. et al. Longitudinal DNA methylation differences precede type 1 diabetes. *Sci. Rep.* **10**, 3721 (2020). 32111940 10.1038/s41598-020-60758-0 PMC7048736

[69] Starskaia, I. et al. Early DNA methylation changes in children developing beta cell autoimmunity at a young age. *Diabetologia* **65**, 844–860 (2022). 35142878 10.1007/s00125-022-05657-x PMC8960578

[70] Dashti, M. et al. Differentially methylated and expressed genes in familial type 1 diabetes. *Sci. Rep.* **12**, 11045 (2022). 35773317 10.1038/s41598-022-15304-5 PMC9247163

[71] Paul, D. S. et al. Increased DNA methylation variability in type 1 diabetes across three immune effector cell types. *Nat. Commun.* **7**, 13555 (2016). 27898055 10.1038/ncomms13555 PMC5141286

[72] Ott, R. Epigenome-wide association study in blood of children born to a mother with or without type 1 diabetes. *Zenodo*10.5281/zenodo.17018103 (2025).

