# Supplementary material for: Blood methylome signatures in children exposed to maternal type 1 diabetes are linked to protection against islet autoimmunity
Source: Nat Metab. 2025 Nov 6;7(11):2236–49. doi: 10.1038/s42255-025-01403-w (PMC12638243; doi:10.1038/s42255-025-01403-w)
Supplement: Supplementary file 1 — Reporting Summary [file 42255_2025_1403_MOESM1_ESM.pdf]

nature portfolio

Corresponding author(s): Sandra Hummel

Last updated by author(s): Sep 1, 2025

Reporting Summary

Nature Portfolio wishes to improve the reproducibility of the work that we publish. This form provides structure for consistency and transparency in reporting. For further information on Nature Portfolio policies, see our Editorial Policies and the Editorial Policy Checklist.

Statistics

For all statistical analyses, confirm that the following items are present in the figure legend, table legend, main text, or Methods section.

n/a	Confirmed	
☐	☒	The exact sample size (*n*) for each experimental group/condition, given as a discrete number and unit of measurement
☐	☒	A statement on whether measurements were taken from distinct samples or whether the same sample was measured repeatedly
☐	☒	The statistical test(s) used AND whether they are one- or two-sided<br>*Only common tests should be described solely by name; describe more complex techniques in the Methods section.*
☐	☒	A description of all covariates tested
☐	☒	A description of any assumptions or corrections, such as tests of normality and adjustment for multiple comparisons
☐	☒	A full description of the statistical parameters including central tendency (e.g. means) or other basic estimates (e.g. regression coefficient) AND variation (e.g. standard deviation) or associated estimates of uncertainty (e.g. confidence intervals)
☐	☒	For null hypothesis testing, the test statistic (e.g. F, t, r) with confidence intervals, effect sizes, degrees of freedom and P value noted<br>*Give P values as exact values whenever suitable.*
☒	☐	For Bayesian analysis, information on the choice of priors and Markov chain Monte Carlo settings
☒	☐	For hierarchical and complex designs, identification of the appropriate level for tests and full reporting of outcomes
☐	☒	Estimates of effect sizes (e.g. Cohen's *d*, Pearson's *r*), indicating how they were calculated

Our web collection on statistics for biologists contains articles on many of the points above.

Software and code

Policy information about availability of computer code

Data collection	No software was used for data collection.
Data analysis	Data analysis was performed using R software (v.4.3.2). Specific R packages were used with standard settings for respective analysis as indicated in the manuscript. Details are given in the Methods section. Customized code is publicly available.

For manuscripts utilizing custom algorithms or software that are central to the research but not yet described in published literature, software must be made available to editors and reviewers. We strongly encourage code deposition in a community repository (e.g. GitHub). See the Nature Portfolio guidelines for submitting code & software for further information.

Data

Policy information about availability of data

All manuscripts must include a data availability statement. This statement should provide the following information, where applicable:

- Accession codes, unique identifiers, or web links for publicly available datasets
- A description of any restrictions on data availability
- For clinical datasets or third party data, please ensure that the statement adheres to our policy

We have included comprehensive summary statistics of all significant results within the main text and supplementary tables. The publicly available data used in this study can be accessed from the HELIX project (https://helixomics.isglobal.org/downloads/downloads.html) and the EWAS catalog repositories (https://

www.ewascatalog.org/download/). Full summary statistics of the meta-EWAS are available at Zenodo (https://doi.org/10.5281/zenodo.17018103). The data that supports the findings of this study are available from the corresponding author upon reasonable request.

Research involving human participants, their data, or biological material

Policy information about studies with human participants or human data. See also policy information about sex, gender (identity/presentation), and sexual orientation and race, ethnicity and racism.

Reporting on sex and gender	We used the term sex conferring to biological sex. Sex was determined based on self-reported questionnaires and confirmed by genetic analysis (samples that had a sex mismatch were excluded from analysis). The distribution of females (n=859, 49%) and males (n=893) in the study cohort is presented in Supplementary Table S1. EWAS analyses were adjusted for age at DNA sample collection and sex. Sex-specific analyses were performed to evaluate potential sex-specific associations.
Reporting on race, ethnicity, or other socially relevant groupings	Unfortunately, we did not collect information on race and ethnicity or other socially relevant grouping variables in our cohorts and were therefore unable to include these variables as potential confounders in the analysis. We discuss this limitation in the manuscript.
Population characteristics	Population characteristics, including age at sample collection and sex, are described in detail in Supplementary Table S1.
Recruitment	For the BABYDIAB/BABYDIET study, children were recruited through a network of collaborating general practitioner pediatricians, diabetes outpatient departments and obstetric departments in Germany. The POInT cohort was recruited through the Global platform for prevention of autoimmune diabetes (GPPAD). GPPAD is a consortium of several European research institutions providing an international infrastructure that enables population-based screening for the identification of newborns and infants who have an increased risk for type 1 diabetes.
Ethics oversight	Bayerische Landesärztekammer, Ludwig-Maximilians University, Munich, Germany; Medical Faculty of Technische Universität München, Germany, the Medical University of Warsaw, Poland, the UK Health Research Authority, UK, Onderzoek UZ/KU Leuven, Belgium, and Regionala Etikprövningsnämnden i Lund, Sweden.

Note that full information on the approval of the study protocol must also be provided in the manuscript.

Field-specific reporting

Please select the one below that is the best fit for your research. If you are not sure, read the appropriate sections before making your selection.

☒ Life sciences ☐ Behavioural & social sciences ☐ Ecological, evolutionary & environmental sciences

For a reference copy of the document with all sections, see nature.com/documents/nr-reporting-summary-flat.pdf

Life sciences study design

All studies must disclose on these points even when the disclosure is negative.

Sample size	The whole study sample includes 1752 children from two prospective cohorts. All children had an increased risk for the development of type 1 diabetes. It has been recommended that a sample size above 1000 is sufficient for epigenome-wide association studies.
Data exclusions	Only children with an available DNA sample with adequate quality and quantity were included for DNA methylation measurements. Samples that failed intensive quality control were excluded from the analysis. We provide flow-charts in the manuscript (Extended Data Fig. 1) to indicate the numbers of available samples and exclusions.
Replication	We meta-analysed data from two independent studies to increase the robustness of findings. A key finding in our analysis was validated in a third independent cohort of children.
Randomization	This study used data from two cohorts. BABYDIAB/BABYDIET are prospective observational cohort studies, POInT is an ongoing randomized controlled trial. Because the POInT trial is still unblinded, we are unable to examine whether the intervention compared with placebo affected the reported associations. Since there is randomization, we expect that offspring of mothers with and without type 1 diabetes are randomly assigned to intervention and placebo groups and would therefore not expect that the intervention affects the reported associations.
Blinding	Investigators were blinded to group allocation (with respect to group allocation in the POInT trial and to maternal type 1 diabetes exposure in utero yes/no).

Reporting for specific materials, systems and methods

We require information from authors about some types of materials, experimental systems and methods used in many studies. Here, indicate whether each material, system or method listed is relevant to your study. If you are not sure if a list item applies to your research, read the appropriate section before selecting a response.

Materials & experimental systems

n/a	Involved in the study
☒	☐ Antibodies
☒	☐ Eukaryotic cell lines
☒	☐ Palaeontology and archaeology
☒	☐ Animals and other organisms
☒	☐ Clinical data
☒	☐ Dual use research of concern
☒	☐ Plants

Methods

n/a	Involved in the study
☒	☐ ChIP-seq
☒	☐ Flow cytometry
☒	☐ MRI-based neuroimaging

Plants

Seed stocks	n/a
Novel plant genotypes	n/a
Authentication	n/a
